# Rationally Designed Minimal Bioactive Domains of AS-48 Bacteriocin Homologs Possess Potent Antileishmanial Properties

**DOI:** 10.1128/spectrum.02658-22

**Published:** 2022-11-07

**Authors:** Hannah N. Corman, Jessica N. Ross, Francisco R. Fields, Douglas A. Shoue, Mary Ann McDowell, Shaun W. Lee

**Affiliations:** a University of Notre Dame, Department of Biological Sciences, Notre Dame, Indiana, USA; b University of Notre Dame, Eck Institute for Global Health, Notre Dame, Indiana, USA; c Johnson & Johnson Consumer Products Inc., Skillman, New Jersey, USA; University of Arkansas for Medical Sciences; University of Central Arkansas

**Keywords:** AS-48, *Leishmania*, antileishmanial, antimicrobial peptides, antiparasitic agents, bacteriocins

## Abstract

Leishmaniasis, a category I neglected tropical disease, is a group of diseases caused by the protozoan parasite *Leishmania* species with a wide range of clinical manifestations. Current treatment options can be highly toxic and expensive, with drug relapse and the emergence of resistance. Bacteriocins, antimicrobial peptides ribosomally produced by bacteria, are a relatively new avenue for potential antiprotozoal drugs. Particular interest has been focused on enterocin AS-48, with previously proven efficacy against protozoan species, including *Leishmania* spp. Sequential characterization of enterocin AS-48 has illustrated that antibacterial bioactivity is preserved in linearized, truncated forms; however, minimal domains of AS-48 bacteriocins have not yet been explored against protozoans. Using rational design techniques to improve membrane penetration activity, we designed peptide libraries using the minimal bioactive domain of AS-48 homologs. Stepwise changes to the charge (*z*), hydrophobicity (*H*), and hydrophobic dipole moment (μ*H*) were achieved through lysine and tryptophan substitutions and the inversion of residues within the helical wheel, respectively. A total of 480 synthetic peptide variants were assessed for antileishmanial activity against Leishmania donovani. One hundred seventy-two peptide variants exhibited 50% inhibitory concentration (IC_50_) values below 20 μM against axenic amastigotes, with 60 peptide variants in the nanomolar range. Nine peptide variants exhibited potent activity against intracellular amastigotes with observed IC_50_ values of <4 μM and limited *in vitro* host cell toxicity, making them worthy of further drug development. Our work demonstrates that minimal bioactive domains of naturally existing bacteriocins can be synthetically engineered to increase membrane penetration against *Leishmania* spp. with minimal host cytotoxicity, holding the promise of novel, potent antileishmanial therapies.

**IMPORTANCE** Leishmaniasis is a neglected tropical disease caused by protozoan parasites of the genus *Leishmania*. There are three primary clinical forms, cutaneous, mucocutaneous, and visceral, with visceral leishmaniasis being fatal if left untreated. Current drug treatments are less than ideal, especially in resource-limited areas, due to the difficult administration and treatment regimens as well as the high cost and the emergence of drug resistance. Identifying potent antileishmanial agents is of the utmost importance. We utilized rational design techniques to synthesize enterocin AS-48 and AS-48-like bacteriocin-based peptides and screened these peptides against L. donovani using a fluorescence-based phenotypic assay. Our results suggest that bacteriocins, specifically these rationally designed AS-48-like peptides, are promising leads for further development as antileishmanial drugs.

## INTRODUCTION

Leishmaniasis is a vector-borne, parasitic disease caused by protozoan parasites of the *Leishmania* genus (1, 2). The disease is endemic in 98 countries, placing almost 300 million people at risk of infection each year (3) and resulting in an estimated 2.4 million disability-adjusted life years (DALYs) (4). Leishmaniasis has a wide spectrum of clinical manifestations, from self-healing skin lesions to hepatosplenomegaly and fatality. There are three primary clinical forms: visceral, cutaneous, and mucocutaneous leishmaniasis (5). Visceral leishmaniasis, the deadliest form, results in a 95% mortality rate if left untreated (6). The World Health Organization (WHO) has declared leishmaniasis a category I neglected tropical disease, and as such, it does not receive adequate attention related to its burden (7).

There are limited viable treatment options currently available for leishmaniasis (8). In resource-limited areas where leishmaniasis is prevalent, pentavalent antimonials are prescribed for all manifestations; however, these compounds are available only intravenously, exhibit high toxicity, and require prolonged treatment, thus resulting in increased clinical and financial burdens (9–11). Furthermore, increasing numbers of cases of drug resistance to pentavalent antimonials have been observed (12, 13). Due to incidents of disease relapse as a result of drug resistance, other agents such as amphotericin B, paromomycin, and miltefosine are being used (14). Unfortunately, these treatment options are costly and have high cellular toxicity, and drug resistance has also been observed (15). There is a great need for new, safe, and effective antileishmanial therapeutics.

Antimicrobial peptides (AMPs) are an intriguing avenue as alternative antiprotozoal agents or to complement current antiprotozoals (16, 17). AMPs are highly diverse peptides, which span the range from prokaryotes to lower and higher eukaryotes (18). Many AMPs are membrane active and have been previously shown to have potent antiparasitic activity (19, 20). Specifically, natural and synthetic cationic AMPs have been shown to be effective against protozoan parasites such as Trypanosoma cruzi (21), *Plasmodium* (22), and *Leishmania* (23). Leishmanicidal activity has been observed with histatin 5, a human salivary AMP, which gains entry to cells via membrane targeting, causing the consequential depolarization of the membrane, and additionally targets mitochondrial ATP synthesis in *Leishmania* spp. (24). Bombinins H2 and H4, isolated and purified from the skin secretions of the frog species Bombina variegata, have also demonstrated potent antileishmanial activity (25, 26). Due to their proven efficacy, high specificity, decreased drug interactions, and low toxicity, AMPs may be untapped sources of novel antileishmanial drug therapies.

Of particular interest in the exploration of novel antimicrobial drugs are bacteriocins, which are AMPs produced by bacteria (27). These ribosomally produced peptides have been shown to have bactericidal (28, 29), fungicidal (30, 31), and parasiticidal (32) properties, giving promise as an alternative to conventional chemical-based antimicrobials. Bacteriocins are characterized into three classes based on size, content, stability, and posttranslational modification (33). Class I (modified) and class II (unmodified) bacteriocins both start as a propeptide containing a leader sequence, which is then cleaved during ribosomal translation, resulting in the functional peptide (34). Posttranslationally, class I has further subgroup classifications based on specific modifications, such as heterocyclization, glycosylation, and head-to-tail circularization. Class III bacteriocins are large molecules of >10 kDa and are thermolabile. Nisin, a class I lantibiotic produced by Lactococcus lactis, has been FDA approved for use as a food preservative (35). Another lantibiotic, GP15cin, is used in ethanol fermentation plants to control inappropriate bacterial growth (36). Bacteriocins are a promising alternative to clinical antimicrobials, as antimicrobial resistance is increasing.

Due to their proteinaceous nature, bacteriocins can be modified via biochemical engineering techniques to investigate bioactive domains and improve specific functions (37). Biochemical engineering techniques to modify AMPs for antimicrobial therapies have been explored using site-directed mutagenesis, *de novo* design, and template-assisted approaches to create synthetic peptide libraries for evaluation (38–40). In order to discern residues that may play a key role in the antibacterial properties of AMPs, unbiased alanine substitutions can be made for every amino acid, building large libraries to screen for antimicrobial activity (41). This technique creates a blueprint of which residues may negatively or positively impact bioactivity. Similarly, the intentional truncation of bioactive peptides may aid in the identification of bioactive regions to further understand areas of interest for future modification (42). The *de novo* design of bacteriocins can be employed to generate random or template-assisted peptides, which can subsequently be assessed computationally using biochemical and biophysical parameters (43). Template-assisted approaches evaluate naturally occurring AMPs to identify biochemically active domains, which can then be used as a template in a rational design approach to optimize bioactivity (44). Rational design is a computational approach that introduces minimal, stepwise changes to an amino acid sequence of a scaffold peptide to improve bioactivity.

Enterocin AS-48, a class I bacteriocin, has been well characterized and is currently being investigated for use as an antimicrobial drug against a large variety of infectious disease agents (45). Enterocin AS-48 is a ribosomally produced, circularized bacteriocin encoded by Enterococcus faecalis subsp. *liquefaciens* strain S-48, which undergoes head-to-tail macrocyclization as a posttranslational modification (46). Efforts to engineer synthetic enterocin AS-48 have been made in order to improve mass production and bioactivity. Previously, segments of the bacteriocin have undergone α-ketoacid-hydroxylamine ligation to chemically produce synthetic enterocin AS-48 in a cyclical form (47). However, due to the ligation process, its bioactivity decreased compared to that of the natural product. Furthermore, using limited proteolysis, linear versions of enterocin AS-48 have been purified (48). Truncation of the linear form identified that the membrane-penetrating activity of enterocin AS-48 is attributed to a specific α-helical region on the circular peptide, which largely contains cationic residues (49). Previously, we utilized specific rational design techniques using AS-48 bacteriocin homologs to design a series of reductive peptide variants of AS-48 optimized for membrane-penetrating bioactivity, which subsequently demonstrated potent antibacterial activity (50). Other studies have shown that full-length enterocin AS-48 induced mitochondrial damage to *Leishmania* promastigotes and also exhibited 50% inhibitory concentration (IC_50_) values against Leishmania pifanoi axenic amastigotes of 10.2 ± 1.2 μM (51). Due to this previously confirmed parasiticidal activity, we hypothesized that AS-48-based minimal domain peptides can also exhibit antileishmanial properties. In this paper, we demonstrate the use of rationally designed minimal AS-48 bacteriocin homologs for antileishmanial drug candidate identification; 9 of the 480 peptides exhibited potent activity against Leishmania donovani intracellular amastigotes with IC_50_ values of less than 4 μM, which is within the target product profile (TPP) established by the Drugs for Neglected Diseases Initiative (DNDi) (50).

## RESULTS

### Peptide library screen and axenic amastigote IC_50_ determination.

**FIG 1:**
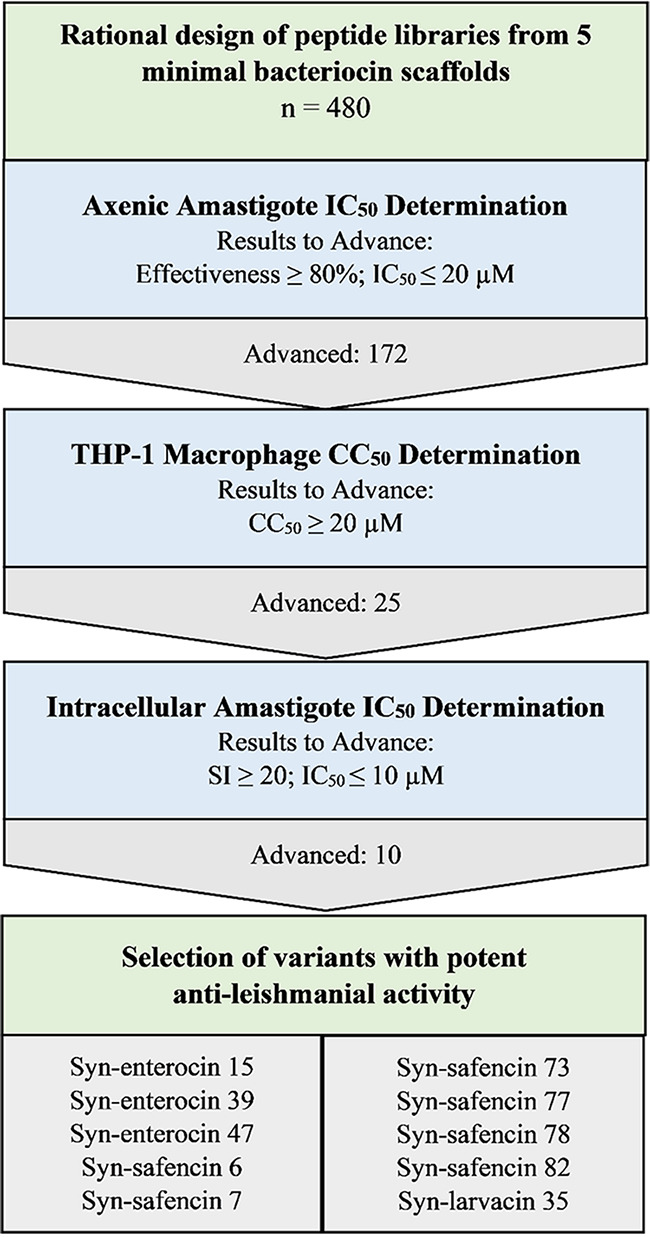
Experimental workflow to determine potent leishmanicidal peptides. Four hundred eighty peptide variants were screened at 20 μM and advanced to the next round if 80% effective compared to the miltefosine control. THP-1 macrophages were used to establish CC_50_ values, subsequently advancing peptides to the next round if the values were above 20 μM. Ten peptide variants found to have an SI value of >20 and an IC_50_ value of <10 μM against axenic amastigotes were tested against intracellular amastigotes.

We utilized a previously developed high-throughput screening assay using transgenic axenic amastigotes expressing a red fluorescent protein, mCherry. Changes in mCherry fluorescence were normalized to 50 μM miltefosine in order to quantify parasite inhibition (52). In total, 5 scaffold 25-mer peptides and 475 synthetic 25-mer peptide variants were screened (Fig. 1). Due to previous data showing that enterocin AS-48 exhibited an IC_50_ value of approximately 20 μM, each peptide was screened at 20 μM to identify minimal AS-48 bacteriocin peptide variants that outperform enterocin AS-48. The assay was repeated, and the average percent parasite killing was determined. A total of 172 peptide variants killed at least 80% of the parasites and were selected for further study. Of the 172 peptide variants that exhibited potent antileishmanial activity, 9 were syn-enterocin, 32 were syn-larvacin, 42 were syn-safencin, 32 were syn-sordellicin, and 57 were syn-xiamencin. Axenic amastigote IC_50_ value determination revealed that 137 variants had IC_50_ values of <5 μM, while 60 of those peptide variants had IC_50_ values in the nanomolar range (see Tables S2 to S6 in the supplemental material).

### Cytotoxicity against THP-1 macrophages.

The 172 peptide variants with antileishmanial activity were then screened against THP-1 macrophages to determine host cell toxicity. The peptides were screened at 20 μM to identify peptide hits, which exhibited IC_50_ values below the previously characterized enterocin AS-48 IC_50_ (51). Twenty-five peptide variants exhibited limited host cell toxicity, with 50% cytotoxic concentration (CC_50_) values above 20 μM and selectivity index (SI) values ranging from 1.40 to 74.07 (Table 1). Of these 25 variants, 4 were syn-enterocin, 3 were syn-larvacin, 10 were syn-safencin, 3 were syn-sordellicin, and 5 were syn-xiamencin. Ten peptide variants, which exhibited CC_50_ values above 20 μM, exhibited axenic amastigote IC_50_ values in the nanomolar range and consequently had an SI of >20. Of these peptide variants, three were syn-enterocin, one was syn-larvacin, and six were syn-safencin. Subsequentially, these 10 peptides were characterized as potential antileishmanial drug candidates and were evaluated further for future clinical use.

**TABLE 1:** Cytotoxicity screening against THP-1 cells with corresponding axenic amastigote IC_50_ values and consequential selectivity index values^*a*^

Peptide variant	THP-1 macrophage CC_50_ (μM)	Axenic amastigotes	
		Mean IC_50_ (μM) ± SD	SI
Syn-enterocin 39	>20	0.27 ± 0.05	74.07
Syn-enterocin 47	>20	0.28 ± 0.07	71.68
Syn-safencin 73	>20	0.35 ± 0.08	56.50
Syn-enterocin 15	>20	0.36 ± 0.03	55.71
Syn-safencin 82	>20	0.375 ± 0.10	53.33
Syn-safencin 77	>20	0.39 ± 0.13	51.28
Syn-safencin 6	>20	0.48 ± 0.07	41.67
Syn-larvacin 35	>20	0.63 ± 0.14	31.95
Syn-safencin 7	>20	0.77 ± 0.08	25.91
Syn-safencin 78	>20	0.94 ± 0.14	21.19
Syn-enterocin 69	>20	1.03 ± 0.13	10.95
Syn-safencin 94	>20	1.17 ± 0.07	17.05
Syn-xiamencin 3	>20	1.26 ± 0.17	15.87
Syn-safencin 2	>20	2.42 ± 0.09	8.26
Syn-xiamencin 41	>20	2.73 ± 0.15	7.33
Syn-xiamencin 43	>20	3.54 ± 0.08	5.65
Syn-safencin 66	>20	3.64 ± 0.10	5.49
Syn-larvacin 14	>20	3.89 ± 0.10	5.14
Syn-larvacin 69	>20	4.14 ± 0.11	4.84
Syn-xiamencin 52	>20	5.23 ± 0.06	3.82
Syn-xiamencin 42	>20	6.83 ± 0.09	2.93
Syn-sordellicin 36	>20	7.59 ± 0.07	2.63
Syn-safencin 72	>20	12.07 ± 0.09	1.66
Syn-sordellicin 19	>20	12.98 ± 0.13	1.54
Syn-sordellicin 6	>20	14.25 ± 0.17	1.40

a Values listed are the means from three replicates.

### Intracellular amastigote IC_50_ determination.

**FIG 2:**
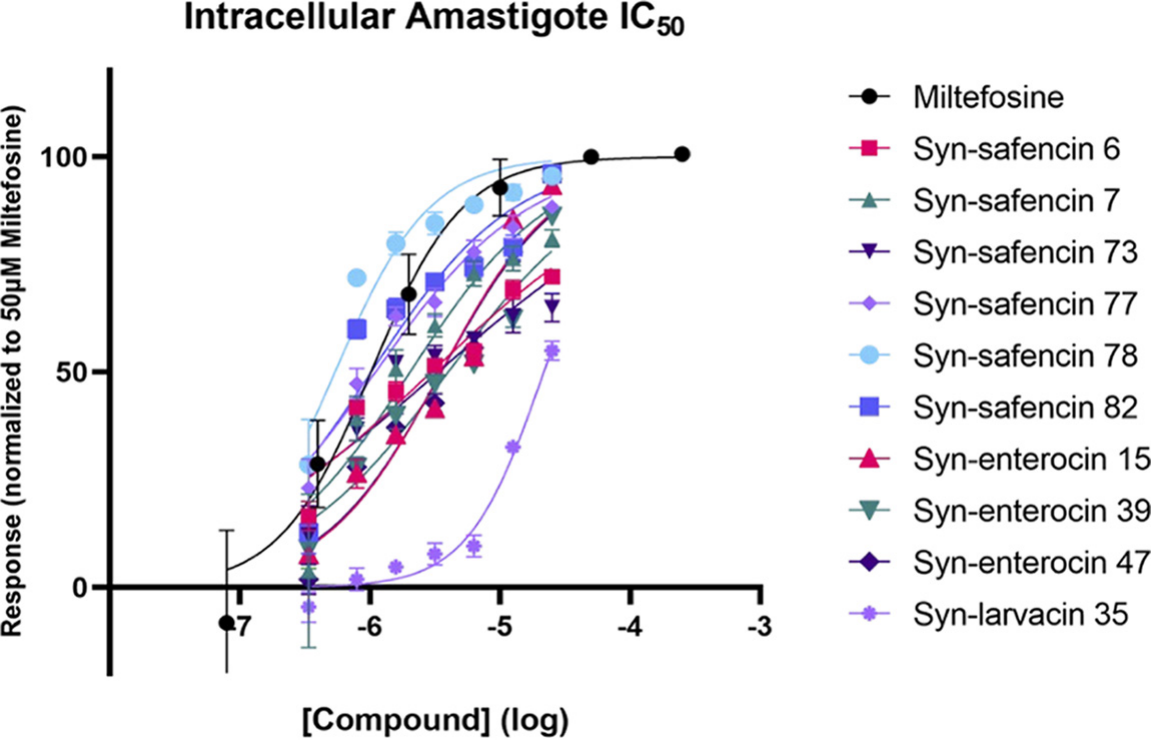
Intracellular amastigote IC_50_ curves of 10 antileishmanial drug candidates. Values listed are the means from three replicates.

As *Leishmania* spp. are obligate intracellular parasites, intracellular amastigote IC_50_ value determination is necessary to understand the clinical relevance of the 10 antileishmanial peptide drug candidates. Initially, peptides were screened against intracellular amastigotes at 20 μM to confirm efficacy, as enterocin AS-48 showed lethality only against promastigotes and axenic amastigotes, with limited activity against intracellular amastigotes. All 10 peptides were observed to have IC_50_ values below 20 μM; thus, IC_50_ values were established against intracellular amastigotes. Nine of the peptides exhibited IC_50_ values at or below 4 μM, including syn-safencin 82, which exhibited potent activity against the intracellular amastigotes, as revealed by an IC_50_ value of 1.0 μM (Fig. 2 and Table 2). Syn-larvacin 35 had an intracellular amastigote IC_50_ value approximately 38 times higher than the initial axenic amastigote IC_50_ value, likely indicating that the peptide was unsuccessful in crossing the macrophage barrier to reach the intracellular parasite.

**TABLE 2:** Intracellular amastigote IC_50_ and selectivity index values^*a*^

Peptide variant	Mean IC_50_ (μM) ± SD	SI
Syn-enterocin 39	4.00 ± 0.04	5.00
Syn-enterocin 47	3.54 ± 0.04	5.66
Syn-safencin 73	3.35 ± 0.08	5.97
Syn-enterocin 15	3.52 ± 0.04	5.69
Syn-safencin 82	1.02 ± 0.07	19.55
Syn-safencin 77	1.59 ± 0.04	12.59
Syn-safencin 6	2.97 ± 0.05	6.74
Syn-larvacin 35	23.67 ± 0.02	0.85
Syn-safencin 7	2.04 ± 0.06	9.82
Syn-safencin 78	3.89 ± 0.06	5.14

a Values listed are the means from three replicates.

### Secondary structure and thermostability analyses.

**FIG 3:**
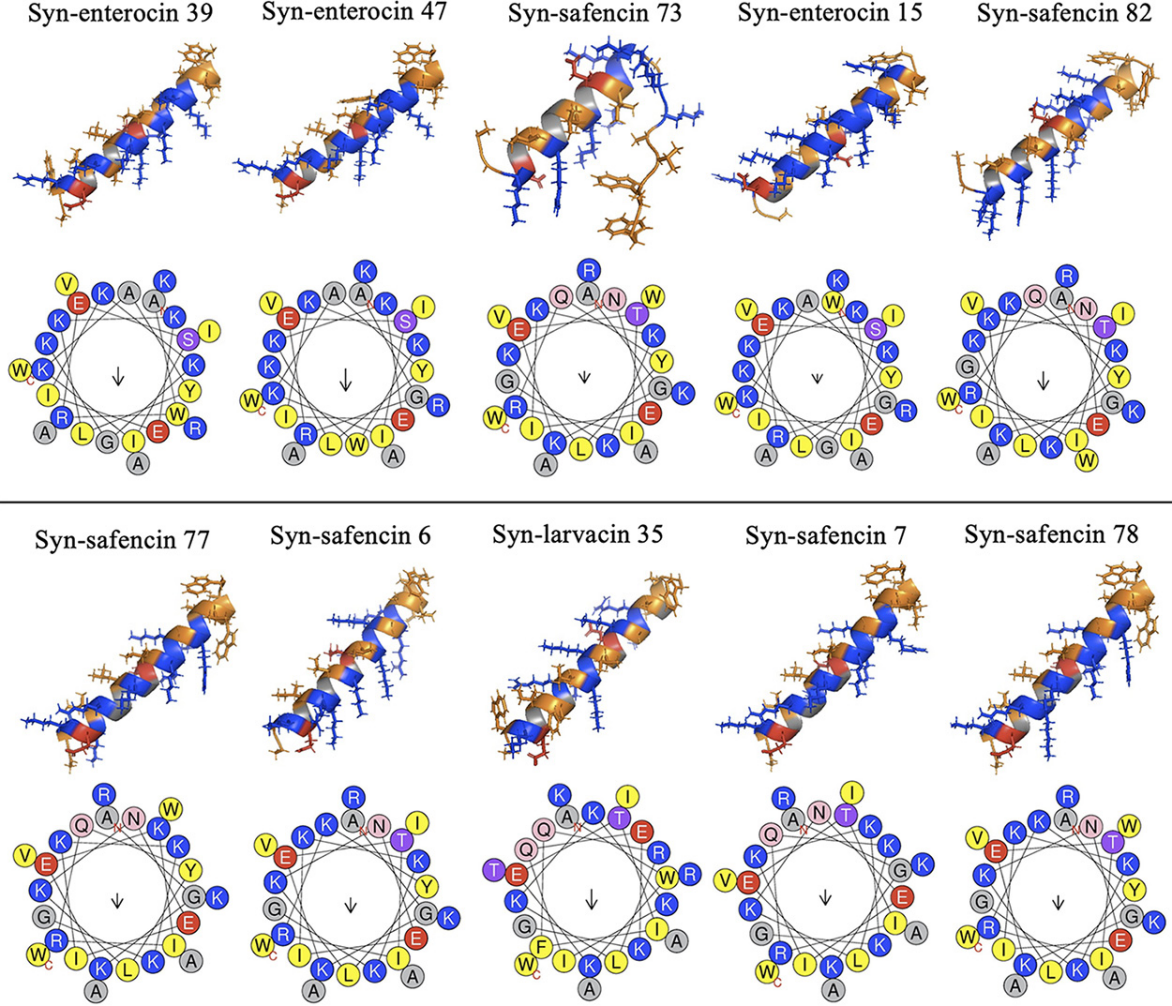
PEPFOLD3 secondary structure predictions for antileishmanial drug candidates, visualized in PyMOL. Hydrophobic regions are in orange, positive regions are in blue, and negative regions are in red. The helical wheel was computationally predicted using HeliQuest. The arrow size indicates the relative hydrophobic moment.

Computational analysis of secondary structures revealed that all 10 antileishmanial drug candidates took an α-helical structure (Fig. 3). Syn-safencin 73 had a decreased α-helical structure, taking the conformation in the fragment window of _3_KETIRQYLKNEIKKK_17_. Helical wheel predictions along with hydrophobic moment (μ*H*) quantification revealed that amphipathicity may not be a favorable biochemical characteristic in antileishmanial peptide design. Within the peptide libraries, peptide variants with increased amphipathicity, achieved through the inversion of residues within the helical wheel and confirmed using HeliQuest, were not initial hits, indicating an axenic amastigote IC_50_ value of >20 μM. Syn-larvacin 35, a clear outlier after the determination of the intracellular amastigote IC_50_ value, was observed to have an increased mean hydrophobicity (*H*) (*H* = 0.206) and a net charge (*z*) (*z* = 6) in the lowest range observed relative to the other peptide variant hits (Table 3). The most potent peptide with the highest SI value from the intracellular amastigote IC_50_ determination assay, syn-safencin 82, was observed to have the highest net charge (*z* = 8). The majority of the antileishmanial peptide drug candidates were observed to have a net charge of 7, including the second most promising drug candidate, syn-safencin 77. Interestingly, syn-safencin 77 was also observed to have the lowest hydrophobicity, at 0.074.

**TABLE 3:** Biochemical characterization and stability analysis of antileishmanial drug candidates

Peptide variant	*z*	*H*	μ*H*	Melting temp (°C)	Δ*G* (kcal/mol)
Syn-enterocin 39	7	0.19	0.242	78.0	−3.8
Syn-enterocin 47	7	0.19	0.288	86.6	−4.5
Syn-safencin 73	6	0.124	0.151	102.1	−4.5
Syn-enterocin 15	7	0.178	0.123	77.0	−2.5
Syn-safencin 82	8	0.17	0.238	83.9	−4
Syn-safencin 77	7	0.074	0.191	70.3	−3.8
Syn-safencin 6	7	0.076	0.194	73.0	−2.7
Syn-larvacin 35	6	0.206	0.259	61.0	−5.2
Syn-safencin 7	7	0.028	0.196	60.0	−2.1
Syn-safencin 78	7	0.094	0.181	74.2	−2.8

Quantification of the melting temperature, used as an indicator of thermostability, revealed a large range of melting temperatures from 60.0°C to 102.1°C (Table 3). The most thermostable antileishmanial drug candidate was observed to be syn-safencin 73, with a melting temperature of 102.1°C. Further analysis of the melting temperatures of all axenic amastigote peptide hits showed the high thermostability of both syn-safencin and syn-sordellicin peptide variants, with many peptides having melting temperatures of >100°C; however, the syn-larvacin peptide variants had decreased thermal stability, as observed by melting temperatures of <60°C. The change in the Gibbs free energy (Δ*G*) was used as an indication of thermodynamic stability, subtracting the relative energies of the folded state (*G_f_*) from those of the unfolded state (*G_u_*); thus, the more negative the value, the more thermodynamically favorable. Thermodynamic stability varied from −2.1 to −5.2 kcal/mol within the 10 antileishmanial drug candidates. Among all initial peptide hits, Δ*G* ranged from −0.4 to −10.6 kcal/mol, indicating large variations in thermodynamic stability.

## DISCUSSION

In this work, we determined the efficacy and cytotoxicity of 480 synthetic, minimal AS-48 bacteriocin homolog peptide variants against L. donovani using phenotypic *in vitro* fluorometric assays. These peptide variants have been shown to have potent antibacterial properties but have not been previously identified as antileishmanial agents. This study illustrates the successful use of rational design using the minimal domain of naturally occurring bacteriocin scaffolds to increase leishmanicidal activity. Specifically, enterocin AS-48 previously exhibited IC_50_ values against axenic amastigotes of 10.2 to 19.5 μM depending on the *Leishmania* species (49). However, with rationally designed minimal AS-48 bacteriocin homologs, we identified 137 minimal peptide variants with IC_50_ values against axenic amastigotes of <5 μM, 60 of which were in the nanomolar range. Importantly, these peptide variants had IC_50_ values similar to those reported previously for AS-48 against Gram-positive bacteria (53–55) and lower IC_50_ values than those of many membrane-active eukaryotic peptides (56, 57). Furthermore, we identified nine variants with IC_50_ values at or below 4 μM against intracellular amastigotes, while enterocin AS-48 was previously shown to have limited activity against the intracellular parasite (49), a more clinically relevant analysis of drug efficacy *in vitro*. The nine antileishmanial peptides also exhibited limited host cell toxicity against THP-1 macrophages; thus, syn-enterocins 15, 39, and 47 and syn-safencins 6, 7, 73, 77, 78, and 82 are worthy of further characterization and development as potential chemotherapeutics.

The peptide variants that we assessed were rationally designed to increase the net charge (*z*), hydrophobicity (*H*), and hydrophobic moment (μ*H*), a quantitative measurement of amphipathicity. These changes were achieved through the replacement of the short-chain amino acids alanine and glycine with lysine, the replacement of aliphatic and nonpolar short-chain amino acids with tryptophan, and the inversion of amino acid residues within the helical wheel. These 3 modifications are present in the nine potent peptide variants. When comparing the final three intracellular IC_50_ syn-enterocin hits, syn-enterocins 15, 39, and 47, to the syn-enterocin scaffold sequence, each peptide variant had amino acids 17 and 18, glycine and lysine, flipped. In addition, each of these peptide variants had a single amino acid replaced with tryptophan; syn-enterocin 15 has a tryptophan at position 1, syn-enterocin 39 has a tryptophan at position 2, and syn-enterocin 47 has a tryptophan at position 17. When comparing the six syn-safencin peptide variants evaluated for intracellular IC_50_ values to the syn-safencin scaffold sequence, a tryptophan substitution toward the C terminus was present in syn-safencins 73, 77, 78, and 82. In addition, five of these syn-safencin peptide variants with potent intracellular activity had amino acids replaced with lysine toward the N terminus, between positions 4 and 9. The replacement of amino acids with tryptophan toward either the N terminus or the C terminus has been shown previously to improve antimicrobial activity (50), consistent with our findings that these key amino acid positions are critical for activity.

The replacement of short-chain amino acids with a positively charged lysine increases the cationic character of the peptide, and this is believed to increase the overall affinity of the peptide for the more anionic target membrane. Based on our observations, modifications toward either terminus of the peptide seem to have a substantial effect on peptide potency against L. donovani. Additionally, our most potent intracellular amastigote drug candidate, syn-safencin 82, had an increased net charge of 8. Comparatively, all other antileishmanial drug candidates exhibited a net charge of either 6 or 7. Interestingly, our computational analysis indicated that amphipathicity may not be an important biochemical property of optimized antileishmanial compounds. This was first illustrated when quantifying the amphipathicity of all peptide variants. Peptides with high amphipathicity did not exhibit antileishmanial properties during our initial screen against axenic amastigotes. Furthermore, all 10 final antileishmanial drug candidates had relatively low hydrophobic dipole moment values, supporting evidence that this biochemical parameter may not optimize membrane-penetrating activity against parasitic targets.

AS-48 homologs and their corresponding truncated, linearized forms are interesting potential chemotherapeutics for many infectious diseases. While we did not investigate the molecular mechanism of action of these peptide variants, the most frequently described model is through membrane disruption (58). The first step in this mechanism of action is initiated by electrostatic attraction between the cationic peptide and anionic phospholipids composing the membrane. Upon electrostatic interactions, peptides may dimerize (59, 60) within the membrane, forming pores (61), ultimately leading to cell death. The differences in the cell membrane lipid composition of the pathogen compared to human host cells is believed to be a key driver of the membrane recognition and selection processes (62). Host macrophages largely contain zwitterionic phospholipids throughout the outer leaflet, potentially preventing substantial electrostatic interactions with cationic peptides that would otherwise cause membrane pore formation and cellular cytotoxicity. This property would allow peptides to potentially be endocytosed in the host cell at concentrations sublytic to the macrophage but would allow the peptide to reach the intracellular parasite to exert activity (63). In addition, the more cationic peptides could preferentially interact with the anionic syndecans or heparan sulfate components found within the extracellular matrix of macrophages for endocytosis. *Leishmania* promastigotes have a surface glycolipid lipophosphoglycan (LPG) that plays critical pleiotropic roles in parasite survival and infectivity in both the sand fly and the mammalian host (64). Previous research has shown that enterocin AS-48 modified the fluidity of protozoal membranes and impaired the activity of several membrane-bound proteins, which provides some support for the potential membrane targeting of our AS-48-based peptide variants (51). However, *Leishmania* amastigotes have significantly less LPG, often corresponding to <100 molecules/cell (65, 66). While amastigotes express little to no LPG, research has shown that various parts of LPG exist as distinct entities, termed glycosylphosphatidylinositol (GPI) antigens (67, 68) or glycosylinositolphospholipids (GIPLs) (65) L. donovani amastigotes have been shown to synthesize GIPLs in quantities comparable to those reported previously for promastigotes (65). These GIPLs are less cationic than the highly cationic LPG, which could decrease the density of the peptides close to the plasma membrane. This information suggests that there could also be intracellular targets that we did not elucidate in this study; previous research proposed *Leishmania* mitochondria as likely targets due to a strong interaction between cardiolipin and AS-48 (51). An intracellular target could also explain the submicromolar efficacy, similar to the efficacy seen against other kinetoplastids like Trypanosoma brucei (32).

AMPs such as enterocin AS-48 have several characteristics that would be advantageous for further development as antileishmanial agents. Bacteriocins can be produced in large amounts at a limited cost (65), are highly stable with a compact structure (66, 67), are resistant to exopeptidase degradation, and have low immunogenicity (68). Our study validates the use of rational design-based approaches to produce minimal domain peptide libraries that can be screened to identify lead peptide candidates with potent antiprotozoal activity and low host cell toxicity. Our library screen identified several minimal AS-48 bacteriocin-based peptides that retained strong leishmanicidal activity against clinically relevant intracellular amastigote forms. Our results prompt further studies to investigate minimal peptides as potent antileishmanial drug candidates, along with further screening of other protozoan parasites.

## MATERIALS AND METHODS

### Rational design and synthesis of AS-48-based antimicrobial peptide libraries.

The bioactive regions of enterocin AS-48 and AS-48 bacteriocin homologs were used as a scaffold for rationally designed antimicrobial peptide libraries as previously described (see Table S1 in the supplemental material) (50). Briefly, the Basic Local Alignment Search Tool (BLAST) was used to identify previously uncharacterized AS-48 bacteriocin homologs produced by Bacillus safensis, Clostridium sordellii, Paenibacillus larvae, and Bacillus xiamenensis. These minimal regions, along with the truncated bioactive region of enterocin AS-48, were then used as a scaffold for library design. Specific rational design strategies were employed for overall library design to optimize antimicrobial properties. First, aliphatic and nonpolar short-chain amino acids were replaced with either lysine or tryptophan, hypothesized to increase peptide electrostatic affinity and penetration into the phospholipid bilayer, respectively (69–71). Next, to increase the amphipathic nature of the peptide, amino acids were inverted within the helical wheel to aggregate hydrophobic amino acids. The clustering of hydrophobic amino acids on the peptide surface may facilitate insertion into the hydrophobic membrane core (72–74). In total, 95 synthetic 25-mer peptide variants of each scaffold peptide, designated syn-enterocin, syn-safencin, syn-sordellicin, syn-larvacin, and syn-xiamencin, were designed based on these defined biophysical parameters and commercially synthesized by GenScript (Piscataway, NJ). Synthesis was confirmed to be >95% pure and was verified by high-performance liquid chromatography–mass spectrometry (HPLC-MS) prior to use (GenScript). Lyophilized peptides were suspended in Nanopure water, diluted to a final stock concentration of 1.28 mM, and stored at −20°C.

### Parasite culture.

Leishmania donovani transgenic strain 1S2D (MHOM/SD/62/1S-CL2d) clone LdB constitutively expressing mCherry51 was cultured in M199 supplemented with 10% fetal calf serum (FCS) at 27°C at pH 7.4. Axenic amastigotes were differentiated as described previously (69).

### Antileishmanial peptide library screen and IC_50_ quantification.

Axenic amastigotes were differentiated as described previously (69); briefly, transgenic L. donovani promastigotes expressing mCherry were differentiated by pH and temperature shifts. Amastigotes were seeded into 96-well plates at 5 × 10^6^ cells/mL and incubated for 48 h in the presence of decreasing drug concentrations, starting at 20 μM, along with the appropriate solvent controls. Fluorescence was monitored (587-nm excitation/610-nm emission) over 48 h using a FlexStation 3 benchtop multimode reader (Molecular Devices). At 50 μM miltefosine, 99% parasite inhibition is observed. Thus, changes in mCherry fluorescence were normalized to the 50 μM miltefosine control in order to quantify parasite inhibition. Each assay was performed in triplicate. The IC_50_ values were calculated by nonlinear regression analysis using GraphPad Prism 9.0 for Windows.

### Evaluation of cytotoxicity against THP-1 macrophages.

THP-1 (human acute monocytic leukemia-derived) host macrophages were cultured in RPMI 1640 supplemented with 10% FCS and a penicillin-streptomycin antibiotic solution (10,000 U/mL penicillin, 10,000 μg/mL streptomycin) at 37°C with 5% CO_2_. THP-1 cells were incubated with 0.25 μM phorbol 12-myristate 7-acetate (PMA) for 48 h to differentiate into mature macrophages. Differentiated macrophages were seeded into 96-well plates at 5 × 10^6^ cells/mL and incubated for 48 h in the presence of 20 μM antimicrobial peptides along with the appropriate solvent controls. Cell viability assays were conducted using CellTiter-Blue (Promega), based on resazurin reduction. After the 48-h incubation period, CellTiter-Blue reagent was added to cells for 4 h at 37°C, and fluorescence was measured (555-nm excitation/580-nm emission) using a FlexStation 3 benchtop multimode reader (Molecular Devices). Each assay was performed in triplicate. The CC_50_ values were calculated by nonlinear regression analysis using GraphPad Prism 9.0 for Windows.

### Intracellular leishmanicidal activity analysis.

Transgenic L. donovani promastigotes constitutively expressing mCherry and THP-1 macrophages were cultured as described above. Metacyclic promastigotes were isolated using a Ficoll-400 density gradient as previously described (70). Differentiated macrophages were exposed to parasites for 4 h at a multiplicity of infection (MOI) of 10 parasites:1 macrophage. After 4 h, the macrophages were washed thoroughly with 1× phosphate-buffered saline (PBS) (pH 7.4) and incubated overnight at 37°C with 5% CO_2_. Macrophages were transferred to 96-well plates at 5 × 10^6^ cells/mL and exposed to antimicrobial peptides at 20 μM. The mCherry fluorescence was monitored (587-nm excitation/610-nm emission) over 48 h using a FlexStation 3 benchtop multimode reader (Molecular Devices). Changes in mCherry fluorescence were normalized to the 50 μM miltefosine control to quantify parasite inhibition, as 50 μM miltefosine corresponds to 99% intracellular parasite inhibition. Each assay was performed in triplicate. The IC_50_ values were calculated by nonlinear regression analysis using GraphPad Prism 9.0 for Windows.

### Computational analysis of secondary structure and stability.

Secondary structure was computationally predicted using TrRosetta and visualized in PyMOL (71, 72). Hydrophobic, positive, and negative regions were colored in orange, blue, and red, respectively. To investigate the hydrophobic dipole moment (μ*H*), which characterizes the amphipathic nature of the peptides, along with the mean hydrophobicity (*H*) and net charge (*z*), and to visualize the predicted helical wheel, HeliQuest was used (73). To analyze stability, the generated .PDB files from TrRosetta were inputted into SCooP, a bioinformatic tool used to predict protein stability curves (74). The melting temperature (degrees Celsius) was used to predict thermostability, while the quantified change in the folding free energy (Δ*G*) was used to determine thermodynamic stability.

## Supplementary Material

Reviewer comments

## References

[1] Elmahallawy EK, Sampedro Martinez A, Rodriguez-Granger J, Hoyos-Mallecot Y, Agil A, Navarro Mari JM, Gutierrez Fernandez J. 2014. Diagnosis of leishmaniasis. J Infect Dev Ctries 8:961–972. doi:10.3855/jidc.4310. 25116660

[2] Steverding D. 2017. The history of leishmaniasis. Parasit Vectors 10:82. doi:10.1186/s13071-017-2028-5. 28202044 PMC5312593

[3] Desjeux P. 2004. Leishmaniasis: current situation and new perspectives. Comp Immunol Microbiol Infect Dis 27:305–318. doi:10.1016/j.cimid.2004.03.004. 15225981

[4] Alvar J, Vélez ID, Bern C, Herrero M, Desjeux P, Cano J, Jannin J, den Boer M, WHO Leishmaniasis Control Team. 2012. Leishmaniasis worldwide and global estimates of its incidence. PLoS One 7:e35671. doi:10.1371/journal.pone.0035671. 22693548 PMC3365071

[5] McGwire BS, Satoskar AR. 2014. Leishmaniasis: clinical syndromes and treatment. QJM 107:7–14. doi:10.1093/qjmed/hct116. 23744570 PMC3869292

[6] Jervis S, Chapman LAC, Dwivedi S, Karthick M, Das A, Le Rutte EA, Courtenay O, Medley GF, Banerjee I, Mahapatra T, Chaudhuri I, Srikantiah S, Hollingsworth TD. 2017. Variations in visceral leishmaniasis burden, mortality and the pathway to care within Bihar, India. Parasit Vectors 10:601. doi:10.1186/s13071-017-2530-9. 29216905 PMC5719561

[7] Mitra AK, Mawson AR. 2017. Neglected tropical diseases: epidemiology and global burden. Tropic Med Infect Dis 2:36. doi:10.3390/tropicalmed2030036. PMC6082091 30270893

[8] De Menezes JPB, Guedes CES, De Oliveira Almeida Petersen AL, Fraga DBM, Veras PST. 2015. Advances in development of new treatment for leishmaniasis. Biomed Res Int 2015:815023. doi:10.1155/2015/815023. 26078965 PMC4442256

[9] Berbert TRN, de Mello TFP, Wolf Nassif P, Mota CA, Silveira AV, Duarte GC, Demarchi IG, Aristides SMA, Lonardoni MVC, Vieira Teixeira JJ, Silveira TGV. 2018. Pentavalent antimonials combined with other therapeutic alternatives for the treatment of cutaneous and mucocutaneous leishmaniasis: a systematic review. Dermatol Res Pract 2018:9014726. doi:10.1155/2018/9014726. 30675152 PMC6323433

[10] Rodrigues BC, Ferreira MF, Barroso DH, da Motta JOC, de Paula CDR, Porto C, Martins SS, Gomes CM, Sampaio RNR. 2020. A retrospective cohort study of the effectiveness and adverse events of intralesional pentavalent antimonials in the treatment of cutaneous leishmaniasis. Int J Parasitol Drugs Drug Resist 14:257–263. doi:10.1016/j.ijpddr.2020.11.002. 33285343 PMC7723996

[11] Frézard F, Demicheli C, Ribeiro RR. 2009. Pentavalent antimonials: new perspectives for old drugs. Molecules 14:2317–2336. doi:10.3390/molecules14072317. 19633606 PMC6254722

[12] Ponte-Sucre A, Gamarro F, Dujardin J-C, Barrett MP, López-Vélez R, García-Hernández R, Pountain AW, Mwenechanya R, Papadopoulou B. 2017. Drug resistance and treatment failure in leishmaniasis: a 21st century challenge. PLoS Negl Trop Dis 11:e0006052. doi:10.1371/journal.pntd.0006052. 29240765 PMC5730103

[13] Croft SL, Sundar S, Fairlamb AH. 2006. Drug resistance in leishmaniasis. Clin Microbiol Rev 19:111–126. doi:10.1128/CMR.19.1.111-126.2006. 16418526 PMC1360270

[14] Sundar S, Singh A. 2018. Chemotherapeutics of visceral leishmaniasis: present and future developments. Parasitology 145:481–489. doi:10.1017/S0031182017002116. 29215329 PMC5984184

[15] Singh N, Kumar M, Singh RK. 2012. Leishmaniasis: current status of available drugs and new potential drug targets. Asian Pac J Trop Med 5:485–497. doi:10.1016/S1995-7645(12)60084-4. 22575984

[16] Mahlapuu M, Håkansson J, Ringstad L, Björn C. 2016. Antimicrobial peptides: an emerging category of therapeutic agents. Front Cell Infect Microbiol 6:194. doi:10.3389/fcimb.2016.00194. 28083516 PMC5186781

[17] Huan Y, Kong Q, Mou H, Yi H. 2020. Antimicrobial peptides: classification, design, application and research progress in multiple fields. Front Microbiol 11:582779. doi:10.3389/fmicb.2020.582779. 33178164 PMC7596191

[18] Wang S, Zeng X, Yang Q, Qiao S. 2016. Antimicrobial peptides as potential alternatives to antibiotics in food animal industry. Int J Mol Sci 17:603. doi:10.3390/ijms17050603. 27153059 PMC4881439

[19] Lacerda AF, Pelegrini PB, De Oliveira DM, Vasconcelos ÉAR, Grossi-de-Sá MF. 2016. Anti-parasitic peptides from arthropods and their application in drug therapy. Front Microbiol 7:91. doi:10.3389/fmicb.2016.00091. 26903970 PMC4742531

[20] Torrent M, Pulido D, Rivas L, Andreu D. 2012. Antimicrobial peptide action on parasites. Curr Drug Targets 13:1138–1147. doi:10.2174/138945012802002393. 22664071

[21] Adade CM, Oliveira IRS, Pais JAR, Souto-Padrón T. 2013. Melittin peptide kills Trypanosoma cruzi parasites by inducing different cell death pathways. Toxicon 69:227–239. doi:10.1016/j.toxicon.2013.03.011. 23562368

[22] Moreira CK, Rodrigues FG, Ghosh A, Varotti FDP, Miranda A, Daffre S, Jacobs-Lorena M, Moreira LA. 2007. Effect of the antimicrobial peptide gomesin against different life stages of Plasmodium spp. Exp Parasitol 116:346–353. doi:10.1016/j.exppara.2007.01.022. 17376436 PMC1978196

[23] Pretzel J, Mohring F, Rahlfs S, Becker K. 2013. Antiparasitic peptides. Adv Biochem Eng Biotechnol 135:157–192. doi:10.1007/10_2013_191. 23615879

[24] Luque-Ortega JR, Hof W, Veerman ECI, Saugar JM, Rivas L. 2008. Human antimicrobial peptide histatin 5 is a cell-penetrating peptide targeting mitochondrial ATP synthesis in Leishmania. FASEB J 22:1817–1828. doi:10.1096/fj.07-096081. 18230684

[25] Simmaco M, Kreil G, Barra D. 2009. Bombinins, antimicrobial peptides from Bombina species. Biochim Biophys Acta 1788:1551–1555. doi:10.1016/j.bbamem.2009.01.004. 19366600

[26] Zhou C, Wang Z, Peng X, Liu Y, Lin Y, Zhang Z, Qiu Y, Jin M, Wang R, Kong D. 2018. Discovery of two bombinin peptides with antimicrobial and anticancer activities from the skin secretion of Oriental fire-bellied toad, Bombina orientalis. Chem Biol Drug Des 91:50–61. doi:10.1111/cbdd.13055. 28636781

[27] Darbandi A, Asadi A, Mahdizade Ari M, Ohadi E, Talebi M, Halaj Zadeh M, Darb Emamie A, Ghanavati R, Kakanj M. 2022. Bacteriocins: properties and potential use as antimicrobials. J Clin Lab Anal 36:e24093. doi:10.1002/jcla.24093. 34851542 PMC8761470

[28] Simons A, Alhanout K, Duval RE. 2020. Bacteriocins, antimicrobial peptides from bacterial origin: overview of their biology and their impact against multidrug-resistant bacteria. Microorganisms 8:639. doi:10.3390/microorganisms8050639. 32349409 PMC7285073

[29] Field D, Cotter PD, Ross RP, Hill C. 2015. Bioengineering of the model lantibiotic nisin. Bioengineered 6:187–192. doi:10.1080/21655979.2015.1049781. 25970137 PMC4601270

[30] de Souza de Azevedo PO, Mendonça CMN, Moreno ACR, Bueno AVI, de Almeida SRY, Seibert L, Converti A, Watanabe I-S, Gierus M, de Souza Oliveira RP. 2020. Antibacterial and antifungal activity of crude and freeze-dried bacteriocin-like inhibitory substance produced by Pediococcus pentosaceus. Sci Rep 10:12291. doi:10.1038/s41598-020-68922-2. 32704020 PMC7378238

[31] Shehata MG, Badr AN, El-Sohaimy SA. 2018. Novel antifungal bacteriocin from Lactobacillus paracasei KC39 with anti-mycotoxigenic properties. Biosci Res 15:4171–4183.

[32] Martínez-García M, Bart J-M, Campos-Salinas J, Valdivia E, Martínez-Bueno M, González-Rey E, Navarro M, Maqueda M, Cebrián R, Pérez-Victoria JM. 2018. Autophagic-related cell death of Trypanosoma brucei induced by bacteriocin AS-48. Int J Parasitol Drugs Drug Resist 8:203–212. doi:10.1016/j.ijpddr.2018.03.002. 29649664 PMC6039360

[33] Cotter PD, Ross RP, Hill C. 2013. Bacteriocins—a viable alternative to antibiotics? Nat Rev Microbiol 11:95–105. doi:10.1038/nrmicro2937. 23268227

[34] Alvarez-Sieiro P, Montalbán-López M, Mu D, Kuipers OP. 2016. Bacteriocins of lactic acid bacteria: extending the family. Appl Microbiol Biotechnol 100:2939–2951. doi:10.1007/s00253-016-7343-9. 26860942 PMC4786598

[35] Shin JM, Gwak JW, Kamarajan P, Fenno JC, Rickard AH, Kapila YL. 2016. Biomedical applications of nisin. J Appl Microbiol 120:1449–1465. doi:10.1111/jam.13033. 26678028 PMC4866897

[36] Pérez-Ramos A, Madi-Moussa D, Coucheney F, Drider D. 2021. Current knowledge of the mode of action and immunity mechanisms of LAB-bacteriocins. Microorganisms 9:2107. doi:10.3390/microorganisms9102107. 34683428 PMC8538875

[37] Fjell CD, Hiss JA, Hancock REW, Schneider G. 2011. Designing antimicrobial peptides: form follows function. Nat Rev Drug Discov 11:37–51. doi:10.1038/nrd3591. 22173434

[38] Perrin BS, Fu R, Cotten ML, Pastor RW. 2016. Simulations of membrane-disrupting peptides II: AMP piscidin 1 favors surface defects over pores. Biophys J 111:1258–1266. doi:10.1016/j.bpj.2016.08.015. 27653484 PMC5034716

[39] Bozovičar K, Bratkovič T. 2019. Evolving a peptide: library platforms and diversification strategies. Int J Mol Sci 21:215. doi:10.3390/ijms21010215. 31892275 PMC6981544

[40] Field D, Cotter PD, Hill C, Ross RP. 2015. Bioengineering lantibiotics for therapeutic success. Front Microbiol 6:1363. doi:10.3389/fmicb.2015.01363. 26640466 PMC4662063

[41] Kers JA, Sharp RE, Muley S, Mayo M, Colbeck J, Zhu Y, DeFusco AW, Park JH, Handfield M. 2018. Blueprints for the rational design of therapeutic mutacin 1140 variants. Chem Biol Drug Des 92:1940–1953. doi:10.1111/cbdd.13365. 30010233

[42] Sánchez-Hidalgo M, Montalbán-López M, Cebrián R, Valdivia E, Martínez-Bueno M, Maqueda M. 2011. AS-48 bacteriocin: close to perfection. Cell Mol Life Sci 68:2845–2857. doi:10.1007/s00018-011-0724-4. 21590312 PMC11115006

[43] Fields FR, Freed SD, Carothers KE, Hamid MN, Hammers DE, Ross JN, Kalwajtys VR, Gonzalez AJ, Hildreth AD, Friedberg I, Lee SW. 2020. Novel antimicrobial peptide discovery using machine learning and biophysical selection of minimal bacteriocin domains. Drug Dev Res 81:43–51. doi:10.1002/ddr.21601. 31483516 PMC9202646

[44] Fields FR, Carothers KE, Balsara RD, Ploplis VA, Castellino FJ, Lee SW. 2018. Rational design of syn-safencin, a novel linear antimicrobial peptide derived from the circular bacteriocin safencin AS-48. J Antibiot (Tokyo) 71:592–600. doi:10.1038/s41429-018-0032-4. 29463889 PMC8887552

[45] González C, Langdon GM, Bruix M, Gálvez A, Valdivia E, Maqueda M, Rico M. 2000. Bacteriocin AS-48, a microbial cyclic polypeptide structurally and functionally related to mammalian NK-lysin. Proc Natl Acad Sci USA 97:11221–11226. doi:10.1073/pnas.210301097. 11005847 PMC17181

[46] Maqueda M, Gálvez A, Bueno MM, Sanchez-Barrena MJ, González C, Albert A, Rico M, Valdivia E. 2004. Peptide AS-48: prototype of a new class of cyclic bacteriocins. Curr Protein Pept Sci 5:399–416. doi:10.2174/1389203043379567. 15544535

[47] Rohrbacher F, Zwicky A, Bode JW. 2017. Chemical synthesis of a homoserine-mutant of the antibacterial, head-to-tail cyclized protein AS-48 by α-ketoacid-hydroxylamine (KAHA) ligation. Chem Sci 8:4051–4055. doi:10.1039/c7sc00789b. 28580120 PMC5434751

[48] Montalbán-López M, Spolaore B, Pinato O, Martínez-Bueno M, Valdivia E, Maqueda M, Fontana A. 2008. Characterization of linear forms of the circular enterocin AS-48 obtained by limited proteolysis. FEBS Lett 582:3237–3242. doi:10.1016/j.febslet.2008.08.018. 18760277

[49] Cruz VL, Ramos J, Melo MN, Martinez-Salazar J. 2013. Bacteriocin AS-48 binding to model membranes and pore formation as revealed by coarse-grained simulations. Biochim Biophys Acta 1828:2524–2531. doi:10.1016/j.bbamem.2013.05.036. 23756777

[50] Ross JN, Fields FR, Kalwajtys VR, Gonzalez AJ, O’Connor S, Zhang A, Moran TE, Hammers DE, Carothers KE, Lee SW. 2020. Synthetic peptide libraries designed from a minimal alpha-helical domain of AS-48-bacteriocin homologs exhibit potent antibacterial activity. Front Microbiol 11:589666. doi:10.3389/fmicb.2020.589666. 33281785 PMC7689250

[51] Abengózar MÁ, Cebrián R, Saugar JM, Gárate T, Valdivia E, Martínez-Bueno M, Maqueda M, Rivas L. 2017. Enterocin AS-48 as evidence for the use of bacteriocins as new leishmanicidal agents. Antimicrob Agents Chemother 61:e02288-16. doi:10.1128/AAC.02288-16. 28167557 PMC5365675

[52] Corman HN, Shoue DA, Norris-Mullins B, Melancon BJ, Morales MA, McDowell MA. 2019. Development of a target-free high-throughput screening platform for the discovery of antileishmanial compounds. Int J Antimicrob Agents 54:496–501. doi:10.1016/j.ijantimicag.2019.07.013. 31323307

[53] Grande Burgos MJ, Pulido RP, Del Carmen López Aguayo M, Gálvez A, Lucas R. 2014. The cyclic antibacterial peptide enterocin AS-48: isolation, mode of action, and possible food applications. Int J Mol Sci 15:22706–22727. doi:10.3390/ijms151222706. 25493478 PMC4284732

[54] Galvez A, Maqueda M, Martinez-Bueno M, Valdivia E. 1991. Permeation of bacterial cells, permeation of cytoplasmic and artificial membrane vesicles, and channel formation on lipid bilayers by peptide antibiotic AS-48. J Bacteriol 173:886–892. doi:10.1128/jb.173.2.886-892.1991. 1702784 PMC207084

[55] Caballero Gómez N, Abriouel H, Grande MJ, Pérez Pulido R, Gálvez A. 2013. Combined treatments of enterocin AS-48 with biocides to improve the inactivation of methicillin-sensitive and methicillin-resistant Staphylococcus aureus planktonic and sessile cells. Int J Food Microbiol 163:96–100. doi:10.1016/j.ijfoodmicro.2013.02.018. 23558192

[56] Vizioli J, Salzet M. 2002. Antimicrobial peptides versus parasitic infections? Trends Parasitol 18:475–476. doi:10.1016/S1471-4922(02)02428-5. 12473356

[57] Rivas L, Luque-Ortega JR, Andreu D. 2009. Amphibian antimicrobial peptides and protozoa: lessons from parasites. Biochim Biophys Acta 1788:1570–1581. doi:10.1016/j.bbamem.2008.11.002. 19046939

[58] Tuerkova A, Kabelka I, Králová T, Sukeník L, Pokorná Š, Hof M, Vácha R. 2020. Effect of helical kink in antimicrobial peptides on membrane pore formation. Elife 9:e47946. doi:10.7554/eLife.47946. 32167466 PMC7069690

[59] Cebrián R, Martínez-Bueno M, Valdivia E, Albert A, Maqueda M, Sánchez-Barrena MJ. 2015. The bacteriocin AS-48 requires dimer dissociation followed by hydrophobic interactions with the membrane for antibacterial activity. J Struct Biol 190:162–172. doi:10.1016/j.jsb.2015.03.006. 25816760

[60] Mesa-Galloso H, Valiente PA, Valdés-Tresanco ME, Epand RF, Lanio ME, Epand RM, Alvarez C, Tieleman DP, Ros U. 2019. Membrane remodeling by the lytic fragment of sticholysinII: implications for the toroidal pore model. Biophys J 117:1563–1576. doi:10.1016/j.bpj.2019.09.018. 31587828 PMC6838749

[61] Brogden KA. 2005. Antimicrobial peptides: pore formers or metabolic inhibitors in bacteria? Nat Rev Microbiol 3:238–250. doi:10.1038/nrmicro1098. 15703760

[62] Shaw JE, Epand RF, Hsu JCY, Mo GCH, Epand RM, Yip CM. 2008. Cationic peptide-induced remodelling of model membranes: direct visualization by in situ atomic force microscopy. J Struct Biol 162:121–138. doi:10.1016/j.jsb.2007.11.003. 18180166

[63] Henderson JM, Waring AJ, Separovic F, Lee KYC. 2016. Antimicrobial peptides share a common interaction driven by membrane line tension reduction. Biophys J 111:2176–2189. doi:10.1016/j.bpj.2016.10.003. 27851941 PMC5113125

[64] Forestier CL, Gao Q, Boons GJ. 2014. Leishmania lipophosphoglycan: how to establish structure-activity relationships for this highly complex and multifunctional glycoconjugate? Front Cell Infect Microbiol 4:193. doi:10.3389/fcimb.2014.00193. 25653924 PMC4301024

[65] Bali V, Panesar PS, Bera MB. 2016. Trends in utilization of agro-industrial byproducts for production of bacteriocins and their biopreservative applications. Crit Rev Biotechnol 36:204–214. doi:10.3109/07388551.2014.947916. 25430892

[66] Cobos ES, Filimonov VV, Gálvez A, Maqueda M, Valdívia E, Martínez JC, Mateo PL. 2001. AS-48: a circular protein with an extremely stable globular structure. FEBS Lett 505:379–382. doi:10.1016/s0014-5793(01)02841-1. 11576532

[67] Sidek NLM, Halim M, Tan JS, Abbasiliasi S, Mustafa S, Ariff AB. 2018. Stability of bacteriocin-like inhibitory substance (BLIS) produced by pediococcus acidilactici kp10 at different extreme conditions. Biomed Res Int 2018:5973484. doi:10.1155/2018/5973484. 30363649 PMC6180926

[68] Behrens HM, Six A, Walker D, Kleanthous C. 2017. The therapeutic potential of bacteriocins as protein antibiotics. Emerg Top Life Sci 1:65–74. doi:10.1042/ETLS20160016. 33525816 PMC7243282

[69] Morita S, Tagai C, Shiraishi T, Miyaji K, Iwamuro S. 2013. Differential mode of antimicrobial actions of arginine-rich and lysine-rich histones against Gram-positive Staphylococcus aureus. Peptides 48:75–82. doi:10.1016/j.peptides.2013.07.025. 23932939

[70] Fimland G, Eijsink VGH, Nissen-Meyer J. 2002. Mutational analysis of the role of tryptophan residues in an antimicrobial peptide. Biochemistry 41:9508–9515. doi:10.1021/bi025856q. 12135373

[71] Nguyen LT, Chau JK, Perry NA, de Boer L, Zaat SAJ, Vogel HJ. 2010. Serum stabilities of short tryptophan- and arginine-rich antimicrobial peptide analogs. PLoS One 5:e12684. doi:10.1371/journal.pone.0012684. 20844765 PMC2937036

[72] Ong ZY, Wiradharma N, Yang YY. 2014. Strategies employed in the design and optimization of synthetic antimicrobial peptide amphiphiles with enhanced therapeutic potentials. Adv Drug Deliv Rev 78:28–45. doi:10.1016/j.addr.2014.10.013. 25453271

[73] Wang C-K, Shih L-Y, Chang KY. 2017. Large-scale analysis of antimicrobial activities in relation to amphipathicity and charge reveals novel characterization of antimicrobial peptides. Molecules 22:2037. doi:10.3390/molecules22112037. 29165350 PMC6150348

[74] Dennison S, Wallace J, Harris F, Phoenix D. 2005. Amphiphilic helical antimicrobial peptides and their structure/function relationships. Protein Pept Lett 12:31–39. doi:10.2174/0929866053406084. 15638801

