# Supplementary material for: Rationally Designed Minimal Bioactive Domains of AS-48 Bacteriocin Homologs Possess Potent Antileishmanial Properties
Source: Microbiol Spectr. 2022 Nov 7;10(6):e02658-22. doi: 10.1128/spectrum.02658-22 (PMC9769502; doi:10.1128/spectrum.02658-22)
Supplement: Supplemental file 1 — Supplemental material. Download spectrum.02658-22-s0001.pdf, PDF file, 1.5 MB [file spectrum.02658-22-s0001.pdf]

```
Syn-enterocin     AGRESIKAYLKKEIKKKGKRAVIAW
Syn-larvacin      AGKETIRQFLKKKIQEKGKRATIAW
Syn-safencin      AGKETIRQYLKNEIKKKGRKAVIAW
Syn-sordellicin   AGRQTIKAYLRREIRKRGRKAVIAW
Syn-xiamencin     AGRQALTLYLKEELRKRGKKAFIAW
                  **::::  :*:.:::::*::* ***
```

Supplementary Table 1. Sequence alignment of the minimal domains of the truncated AS-48 bacteriocin homolog scaffolds.

Supplementary Table 2. Secondary screening results of Syn-enterocin peptide library. Peptide sequence images were made using Geneious R6 version 6.1.8 for Windows. Syn-enterocin-1 sequence is in full color, while syn-enterocin-2 through -96 only have colored amino acids that differ from the sequence of syn-enterocin-1. The highest concentration screened was 20μM. The values listed are the means of three replicates ± standard deviation (μM). Selectivity index (SI) is calculated as mean CC_{50}/IC_{50}. Axenic amastigote IC_{50} values were determined if below 20μM and those peptides were then screened against THP-1 macrophages. Cytotoxicity CC_{50} values were only determined if the peptide was effective against axenic amastigotes. Due to limited peptide availability, only the 10 peptide candidates with axenic amastigote SI values >20 were then screened in the intracellular amastigote model.

Name	Sequence	THP-1	Axenic Amastigotes		Intracellular Amastigotes	
		CC_{50} (μM)	IC_{50} (μM)	SI	IC_{50} (μM)	SI
Syn-enterocin-1	AGRESIKAYLKKEIKKKGKRAVIAW	<20	19.28 ± 0.11	1.04		
Syn-enterocin-2	AGSERIKAYLKKEIKKKGKRAVIAW					
Syn-enterocin-3	AGRESIAKYLKKEIKKKGKRAVIAW					
Syn-enterocin-4	AGRESIKAELKKYIKKKGKRAVIAW					
Syn-enterocin-5	AGRESIKAYLKKEIKKKGKVARIAW					
Syn-enterocin-6	AGRESIKAYLKKEIKKKGKRIVAAW					
Syn-enterocin-7	AGRESIKAYLKKEIKKGKKRAVIAW					
Syn-enterocin-8	AGSERIAKELKKYIKKGKKVIRAAW					
Syn-enterocin-9	WGRESIKAYLKKEIKKKGKRAVIAW					
Syn-enterocin-10	WGSERIKAYLKKEIKKKGKRAVIAW					
Syn-enterocin-11	WGRESIAKYLKKEIKKKGKRAVIAW					
Syn-enterocin-12	WGRESIKAELKKYIKKKGKRAVIAW					
Syn-enterocin-13	WGRESIKAYLKKEIKKKGKVARIAW					
Syn-enterocin-14	WGRESIKAYLKKEIKKKGKRIVAAW					
Syn-enterocin-15	WGRESIKAYLKKEIKKGKKRAVIAW	>20	0.36 ± 0.03	55.71	3.52 ± 0.04	5.69
Syn-enterocin-16	WGSERIAKELKKYIKKGKKVIRAAW					
Syn-enterocin-17	AGRESIKWYLKKEIKKKGKRAVIAW					
Syn-enterocin-18	AGSERIKWYLKKEIKKKGKRAVIAW					
Syn-enterocin-19	AGRESIWKYLKKEIKKKGKRAVIAW	<20	0.73 ± 0.09	27.25		
Syn-enterocin-20	AGRESIKWELKKYIKKKGKRAVIAW					
Syn-enterocin-21	AGRESIKWYLKKEIKKKGKVARIAW					
Syn-enterocin-22	AGRESIKWYLKKEIKKKGKRIVAAW					
Syn-enterocin-23	AGRESIKWYLKKEIKKGKKRAVIAW					
Syn-enterocin-24	AGSERIWKELKKYIKKGKKVIRAAW					
Syn-enterocin-25	AGREWIKAYLKKEIKKKGKRAVIAW					
Syn-enterocin-26	AGWERIKAYLKKEIKKKGKRAVIAW					
Syn-enterocin-27	AGREWIAKYLKKEIKKKGKRAVIAW					
Syn-enterocin-28	AGREWIKAELKKYIKKKGKRAVIAW					
Syn-enterocin-29	AGREWIKAYLKKEIKKKGKVARIAW					
Syn-enterocin-30	AGREWIKAYLKKEIKKKGKRIVAAW					

Minimal AS-48 bacteriocin-based peptide variants exhibit leishmanicidal activity
Corman et al

Syn-enterocin-31	AGREWIKAYLKKEIKKGKKRAVIAW					
Syn-enterocin-32	AGWERIAKELKKYIKKGKKVIRAAW					
Syn-enterocin-33	AWRESIKAYLKKEIKKKGKRAVIAW					
Syn-enterocin-34	AWSERIKAYLKKEIKKKGKRAVIAW					
Syn-enterocin-35	AWRESIAKYLKKEIKKKGKRAVIAW					
Syn-enterocin-36	AWRESIKAELKKYIKKKGKRAVIAW	<20	3.21 ± 0.07	6.23		
Syn-enterocin-37	AWRESIKAYLKKEIKKKGKVARIAW					
Syn-enterocin-38	AWRESIKAYLKKEIKKKGKRIVAAW					
Syn-enterocin-39	AWRESIKAYLKKEIKKGKKRAVIAW	>20	0.27 ± 0.05	74.07	4.00 ± 0.04	5.00
Syn-enterocin-40	AWSERIAKELKKYIKKGKKVIRAAW					
Syn-enterocin-41	AGRESIKAYLKKEIKKKWKRAVIAW					
Syn-enterocin-42	AGSERIKAYLKKEIKKKWKRAVIAW					
Syn-enterocin-43	AGRESIAKYLKKEIKKKWKRAVIAW					
Syn-enterocin-44	AGRESIKAELKKYIKKKWKRAVIAW					
Syn-enterocin-45	AGRESIKAYLKKEIKKKWKVARIAW					
Syn-enterocin-46	AGRESIKAYLKKEIKKKWKRIVAAW					
Syn-enterocin-47	AGRESIKAYLKKEIKKWKKRAVIAW	>20	0.28 ± 0.07	71.68	3.54 ± 0.04	5.66
Syn-enterocin-48	AGSERIAKELKKYIKKWKKVIRAAW	<20	1.21 ± 0.05	16.49		
Syn-enterocin-49	AGRESIKAYLKKEIKKKGKRAVIWW					
Syn-enterocin-50	AGSERIKAYLKKEIKKKGKRAVIWW					
Syn-enterocin-51	AGRESIAKYLKKEIKKKGKRAVIWW					
Syn-enterocin-52	AGRESIKAELKKYIKKKGKRAVIWW					
Syn-enterocin-53	AGRESIKAYLKKEIKKKGKVARIWW					
Syn-enterocin-54	AGRESIKAYLKKEIKKKGKRIVAWW					
Syn-enterocin-55	AGRESIKAYLKKEIKKGKKRAVIWW					
Syn-enterocin-56	AGSERIAKELKKYIKKGKKVIRAWW					
Syn-enterocin-57	AGRESIKAYLKKEIKKKGKRAWIAW					
Syn-enterocin-58	AGSERIKAYLKKEIKKKGKRAWIAW					
Syn-enterocin-59	AGRESIAKYLKKEIKKKGKRAWIAW					
Syn-enterocin-60	AGRESIKAELKKYIKKKGKRAWIAW	<20	0.29 ± 0.07	68.49		
Syn-enterocin-61	AGRESIKAYLKKEIKKKGKWARIAW					
Syn-enterocin-62	AGRESIKAYLKKEIKKKGKRIWAAW					
Syn-enterocin-63	AGRESIKAYLKKEIKKGKKRAWIAW					
Syn-enterocin-64	AGSERIAKELKKYIKKGKKWIRAAW	<20	0.42 ± 0.13	48.08		
Syn-enterocin-65	AGRESIKKYLKKEIKKKGKRAVIAW					
Syn-enterocin-66	AGSERIKKYLKKEIKKKGKRAVIAW					
Syn-enterocin-67	AGRESIKKYLKKEIKKKGKRAVIAF					
Syn-enterocin-68	AGRESIKKELKKYIKKKGKRAVIAW					
Syn-enterocin-69	AGRESIKKYLKKEIKKKGKVARIAW	>20	1.03 ± 0.13	19.49		
Syn-enterocin-70	AGRESIKKYLKKEIKKKGKRIVAAW					
Syn-enterocin-71	AGRESIKKYLKKEIKKGKKRAVIAW					

Syn-enterocin-72	AGSERIKKELKKYIKKGKKVIRAAW					
Syn-enterocin-73	AGREKIKAYLKKEIKKKGKRAVIAW					
Syn-enterocin-74	AGKERIKAYLKKEIKKKGKRAVIAW					
Syn-enterocin-75	AGREKIAKYLKKEIKKKGKRAVIAW					
Syn-enterocin-76	AGREKIKAELKKYIKKKGKRAVIAW					
Syn-enterocin-77	AGREKIKAYLKKEIKKKGKVARIAW					
Syn-enterocin-78	AGREKIKAYLKKEIKKKGKRIVAAW					
Syn-enterocin-79	AGREKIKAYLKKEIKKGKKRAVIAW					
Syn-enterocin-80	AGKERIAKELKKYIKKGKKVIRAAW					
Syn-enterocin-81	AGRESIKAYLKKEIKKKKKRAVIAW					
Syn-enterocin-82	AGSERIKAYLKKEIKKKKKRAVIAW					
Syn-enterocin-83	AGRESIAKYLKKEIKKKKKRAVIAW					
Syn-enterocin-84	AGRESIKAELKKYIKKKKKRAVIAW					
Syn-enterocin-85	AGRESIKAYLKKEIKKKKKVARIAW					
Syn-enterocin-86	AGRESIKAYLKKEIKKKKKRIVAAW					
Syn-enterocin-87	AGRESIKAYLKKEIKKRKKRAVIAW					
Syn-enterocin-88	AGSERIAKELKKYIKKKKKVIRAAW					
Syn-enterocin-89	AKRESIKAYLKKEIKKKGKRAVIAW					
Syn-enterocin-90	AKSERIKAYLKKEIKKKGKRAVIAW					
Syn-enterocin-91	AKRESIAKYLKKEIKKKGKRAVIAW					
Syn-enterocin-92	AKRESIKAELKKYIKKKGKRAVIAW					
Syn-enterocin-93	AKRESIKAYLKKEIKKKGKVARIAW					
Syn-enterocin-94	AKRESIKAYLKKEIKKKGKRIVAAW					
Syn-enterocin-95	AKRESIKAYLKKEIKKGKKRAVIAW					
Syn-enterocin-96	AKSERIAKELKKYIKKGKKVIRAAW					

Supplementary Table 3. Secondary screening results of Syn-larvacin peptide library. Peptide sequence images were made using Geneious R6 version 6.1.8 for Windows. Syn-larvacin-1 sequence is in full color, while syn-larvacin-2 through -96 only have colored amino acids that differ from the sequence of syn-larvacin-1. The highest concentration screened was 20μM. The values listed are the means of three replicates ± standard deviation (μM). Selectivity index (SI) is calculated as mean CC_{50}/IC_{50}. Axenic amastigote IC_{50} values were determined if below 20μM and those peptides were then screened against THP-1 macrophages. Cytotoxicity CC_{50} values were only determined if the peptide was effective against axenic amastigotes. Due to limited peptide availability, only the 10 peptide candidates with axenic amastigote SI values >20 were then screened in the intracellular amastigote model.

Name	Sequence	THP-1	Axenic Amastigotes		Intracellular Amastigotes	
		CC_{50} (μM)	IC_{50} (μM)	SI	IC_{50} (μM)	SI
Syn-larvacin-1	AGKETIRQFLKKKIQEKGKRATIAW		>20			
Syn-larvacin-2	AGTEKIRQFLKKKIQEKGKRATIAW					
Syn-larvacin-3	AGKETIFQRLKKKIQEKGKRATIAW	<20	0.36 ± 0.08	55.10		
Syn-larvacin-4	AGKETIRQFLKKKIQEGKKRATIAW	<20	1.09 ± 0.09	18.35		
Syn-larvacin-5	AGKETIRQFLKKTIQEKGKRAKIAW					
Syn-larvacin-6	AGKETIRQFLKKKIQEKGKIATRAW					
Syn-larvacin-7	AGTEKIFQRLKKTIQEGKKIAKRAW	<20	5.61 ± 0.16	3.56		
Syn-larvacin-8	AGTEKIFNRLKKTINEGKKIAKRAW					
Syn-larvacin-9	WGKETIRQFLKKKIQEKGKRATIAW					
Syn-larvacin-10	WGTEKIRQFLKKKIQEKGKRATIAW	<20	0.27 ± 0.05	74.35		
Syn-larvacin-11	WGKETIFQRLKKKIQEKGKRATIAW	<20	0.66 ± 0.10	30.30		
Syn-larvacin-12	WGKETIRQFLKKKIQEGKKRATIAW					
Syn-larvacin-13	WGKETIRQFLKKTIQEKGKRAKIAW					
Syn-larvacin-14	WGKETIRQFLKKKIQEKGKIATRAW	>20	3.89 ± 0.10	5.14		
Syn-larvacin-15	WGTEKIFQRLKKTIQEGKKIAKRAW		0.26 ± 0.12			
Syn-larvacin-16	WGTEKIFNRLKKTINEGKKIAKRAW					
Syn-larvacin-17	AGKEWIRQFLKKKIQEKGKRATIAW					
Syn-larvacin-18	AGWEKIRQFLKKKIQEKGKRATIAW					

Syn-larvacin-19	AGKEWIFQRLKKKIQEKGKRATIAW	<20	0.73 ± 0.08	27.43		
Syn-larvacin-20	AGKEWIRQFLKKKIQEGKKRATIAW					
Syn-larvacin-21	AGKEWIRQFLKKTIQEKGKRAKIAW					
Syn-larvacin-22	AGKEWIRQFLKKKIQEKGKIATRAW					
Syn-larvacin-23	AGWEKIFQRLKKTIQEGKKIAKRAW					
Syn-larvacin-24	AGWEKIFNRLKKTINEGKKIAKRAW					
Syn-larvacin-25	AGKETIRQFLKKKIQEKGKRAWIAW					
Syn-larvacin-26	AGTEKIRQFLKKKIQEKGKRAWIAW					
Syn-larvacin-27	AGKETIFQRLKKKIQEKGKRAWIAW					
Syn-larvacin-28	AGKETIRQFLKKKIQEGKKRAWIAW					
Syn-larvacin-29	AGKETIRQFLKKWIQEKGKRAKIAW					
Syn-larvacin-30	AGKETIRQFLKKKIQEKGKIAWRAW					
Syn-larvacin-31	AGTEKIFQRLKKWIQEGKKIAKRAW	<20	3.80 ± 0.13	27.43		
Syn-larvacin-32	AGTEKIFNRLKKWINEGKKIAKRAW	<20	0.23 ± 0.18	86.58		
Syn-larvacin-33	AWKETIRQFLKKKIQEKGKRATIAW					
Syn-larvacin-34	AWTEKIRQFLKKKIQEKGKRATIAW					
Syn-larvacin-35	AWKETIFQRLKKKIQEKGKRATIAW	>20	0.63 ± 0.14	31.95	23.67 ± 0.02	0.85
Syn-larvacin-36	AWKETIRQFLKKKIQEGKKRATIAW		5.48 ± 0.12			
Syn-larvacin-37	AWKETIRQFLKKTIQEKGKRAKIAW	<20	0.29 ± 0.11	69.69		
Syn-larvacin-38	AWKETIRQFLKKKIQEKGKIATRAW					
Syn-larvacin-39	AWTEKIFQRLKKTIQEGKKIAKRAW	<20	1.43 ± 0.10	14.00		
Syn-larvacin-40	AWTEKIFNRLKKTINEGKKIAKRAW	<20	8.35 ± 0.09	2.39		
Syn-larvacin-41	AGKETIRQFLKKKIQEKWKRATIAW					
Syn-larvacin-42	AGTEKIRQFLKKKIQEKWKRATIAW					
Syn-larvacin-43	AGKETIFQRLKKKIQEKWKRATIAW	<20	0.22 ± 0.06	89.69		
Syn-larvacin-44	AGKETIRQFLKKKIQEWKKRATIAW	<20	0.47 ± 0.11	42.74		
Syn-larvacin-45	AGKETIRQFLKKTIQEKWKRAKIAW					
Syn-larvacin-46	AGKETIRQFLKKKIQEKWKIATRAW					

Minimal AS-48 bacteriocin-based peptide variants exhibit leishmanicidal activity
Corman et al

Syn-larvacin-47	AGTEKIFQRLKKTIQEWKKIAKRAW	<20	1.10 ± 0.06	18.26		
Syn-larvacin-48	AGTEKIFNRLKKTINEWKKIAKRAW		0.24 ± 0.07			
Syn-larvacin-49	AGKETIRWFLKKKIWEKGKRATIAW					
Syn-larvacin-50	AGTEKIRWFLKKKIWEKGKRATIAW					
Syn-larvacin-51	AGKETIFWRLKKKIWEKGKRATIAW	<20	0.42 ± 0.12	47.39		
Syn-larvacin-52	AGKETIRWFLKKKIWEGKKRATIAW	<20	1.86 ± 0.14	10.76		
Syn-larvacin-53	AGKETIRWFLKKTIWEKGKRAKIAW					
Syn-larvacin-54	AGKETIRWFLKKKIWEKGKIATRAW					
Syn-larvacin-55	AGTEKIFWRLKKTIWEGKKIAKRAW					
Syn-larvacin-56	AGTEKIFFRLKKTIFEGKKIAKRAW					
Syn-larvacin-57	AGKETIRQFLKKKIQEKGKRWTIAW					
Syn-larvacin-58	AGTEKIRQFLKKKIQEKGKRWTIAW					
Syn-larvacin-59	AGKETIFQRLKKKIQEKGKRWTIAW					
Syn-larvacin-60	AGKETIRQFLKKKIQEGKKRWTIAW					
Syn-larvacin-61	AGKETIRQFLKKTIQEKGKRWKIAW					
Syn-larvacin-62	AGKETIRQFLKKKIQEKGKIWTRAW					
Syn-larvacin-63	AGTEKIFQRLKKTIQEGKKIWKRAW					
Syn-larvacin-64	AGTEKIFNRLKKTINEGKKIWKRAW					
Syn-larvacin-65	AGKETIRQFLKKKIQEKGKRATIWW	<20	0.31 ± 0.07	63.90		
Syn-larvacin-66	AGTEKIRQFLKKKIQEKGKRATIWW					
Syn-larvacin-67	AGKETIFQRLKKKIQEKGKRATIWW					
Syn-larvacin-68	AGKETIRQFLKKKIQEGKKRATIWW	<20	1.31 ± 0.06	15.33		
Syn-larvacin-69	AGKETIRQFLKKTIQEKGKRAKIWW	>20	4.14 ± 0.11	4.84		
Syn-larvacin-70	AGKETIRQFLKKKIQEKGKIATRWW	<20	0.30 ± 0.12	66.45		
Syn-larvacin-71	AGTEKIFQRLKKTIQEGKKIAKRWW					
Syn-larvacin-72	AGTEKIFNRLKKTINEGKKIAKRWW		1.01 ± 0.16			
Syn-larvacin-73	AGKEKIRQFLKKKIQEKGKRATIAW	<20	0.39 ± 0.07	51.81		
Syn-larvacin-74	AGKERIRQFLKKKIQEKGKRATIAW					

Syn-larvacin-75	AGKEKIFQRLKKKIQEKGKRATIAW					
Syn-larvacin-76	AGKEKIRQFLKKKIQEGKKRATIAW	<20	1.58 ± 0.08	12.68		
Syn-larvacin-77	AGKEKIRQFLKKTIQEKGKRAKIAW	<20	10.32 ± 0.16	1.94		
Syn-larvacin-78	AGKEKIRQFLKKKIQEKGKIATRAW					
Syn-larvacin-79	AGKEKIFQRLKKTIQEGKKIAKRAW					
Syn-larvacin-80	AGKEKIFNRLKKTINEGKKIAKRAW					
Syn-larvacin-81	AGKETIRQFLKKKIQEKKKRATIAW	<20	0.26 ± 0.16	76.05		
Syn-larvacin-82	AGTEKIRQFLKKKIQEKKKRATIAW					
Syn-larvacin-83	AGKETIFQRLKKKIQEKKKRATIAW					
Syn-larvacin-84	AGKETIRQFLKKKIQERKKRATIAW					
Syn-larvacin-85	AGKETIRQFLKKTIQEKKKRAKIAW					
Syn-larvacin-86	AGKETIRQFLKKKIQEKKKIATRAW					
Syn-larvacin-87	AGTEKIFQRLKKTIQEKKKIAKRAW					
Syn-larvacin-88	AGTEKIFNRLKKTINEKKKIAKRAW					
Syn-larvacin-89	AGKETIRQFLKKKIQEKGKRAKIAW					
Syn-larvacin-90	AGTEKIRQFLKKKIQEKGKRAKIAW					
Syn-larvacin-91	AGKETIFQRLKKKIQEKGKRAKIAW					
Syn-larvacin-92	AGKETIRQFLKKKIQEGKKRAKIAW					
Syn-larvacin-93	AGKETIRQFLKKRIQEKGKRAKIAW	<20	2.46 ± 0.17	8.13		
Syn-larvacin-94	AGKETIRQFLKKKIQEKGKIAKRAW	<20	18.23 ± 0.19	1.10		
Syn-larvacin-95	AGTEKIFQRLKKKIQEGKKIAKRAW					
Syn-larvacin-96	AGTEKIFNRLKKKINEGKKIAKRAW					

Supplementary Table 4. Secondary screening results of Syn-safencin peptide library. Peptide sequence images were made using Geneious R6 version 6.1.8 for Windows. Syn-safencin-1 sequence is in full color, while syn-safencin-2 through -96 only have colored amino acids that differ from the sequence of syn-safencin-1. The highest concentration screened was 20µM. The values listed are the means of three replicates ± standard deviation (µM). Selectivity index (SI) is calculated as mean CC_{50}/IC_{50}. Axenic amastigote IC_{50} values were determined if below 20µM and those peptides were then screened against THP-1 macrophages. Cytotoxicity CC_{50} values were only determined if the peptide was effective against axenic amastigotes. Due to limited peptide availability, only the 10 peptide candidates with axenic amastigote SI values >20 were then screened in the intracellular amastigote model.

Name	Sequence	THP-1	Axenic Amastigotes		Intracellular Amastigotes	
		CC_{50} (µM)	IC_{50} (µM)	SI	IC_{50} (µM)	SI
Syn-safencin-1	AGKETIRQYLKNEIKKKGRKAVIAW		>20			
Syn-safencin-2	AGKKTIRQYLKNEIKKKGRKAVIAW	>20	2.42 ± 0.09	8.26		
Syn-safencin-3	AGKETIRQYLKNKIKKKGRKAVIAW					
Syn-safencin-4	AGKKTIRQYLKNKIKKKGRKAVIAW					
Syn-safencin-5	AGKEKIRQYLKNEIKKKGRKAVIAW					
Syn-safencin-6	AGKETIRKYLKNEIKKKGRKAVIAW	>20	0.48 ± 0.07	41.67	2.97 ± 0.05	6.74
Syn-safencin-7	AGKETIRQKLKNEIKKKGRKAVIAW	>20	0.77 ± 0.08	25.91	2.04 ± 0.06	9.82
Syn-safencin-8	AGKEKIRKKLKNEIKKKGRKAVIAW					
Syn-safencin-9	WGKETIRQYLKNEIKKKGRKAVIAW					
Syn-safencin-10	WGKKTIRQYLKNEIKKKGRKAVIAW	<20	2.97 ± 0.09	6.75		
Syn-safencin-11	WGKETIRQYLKNKIKKKGRKAVIAW	<20	12.15 ± 0.11	1.65		
Syn-safencin-12	WGKKTIRQYLKNKIKKKGRKAVIAW					
Syn-safencin-13	WGKEKIRQYLKNEIKKKGRKAVIAW					
Syn-safencin-14	WGKETIRKYLKNEIKKKGRKAVIAW	<20	0.67 ± 0.06	30.08		
Syn-safencin-15	WGKETIRQKLKNEIKKKGRKAVIAW	<20	1.71 ± 0.09	11.69		
Syn-safencin-16	WGKEKIRKKLKNEIKKKGRKAVIAW					
Syn-safencin-17	AWKETIRQYLKNEIKKKGRKAVIAW					

Syn-safencin-18	AWKKTIRQYLKNEIKKKGRKAVIAW	<20	7.96 ± 0.14	2.51		
Syn-safencin-19	AWKETIRQYLKNKIKKKGRKAVIAW	<20	0.38 ± 0.08	53.19		
Syn-safencin-20	AWKKTIRQYLKNKIKKKGRKAVIAW					
Syn-safencin-21	AWKEKIRQYLKNEIKKKGRKAVIAW					
Syn-safencin-22	AWKETIRKYLKNEIKKKGRKAVIAW	<20	1.18 ± 0.11	16.89		
Syn-safencin-23	AWKETIRQKLKNEIKKKGRKAVIAW					
Syn-safencin-24	AWKEKIRKKLKNEIKKKGRKAVIAW					
Syn-safencin-25	AGKETWRQYLKNEIKKKGRKAVIAW					
Syn-safencin-26	AGKKTWRQYLKNEIKKKGRKAVIAW					
Syn-safencin-27	AGKETWRQYLKNKIKKKGRKAVIAW					
Syn-safencin-28	AGKKTWRQYLKNKIKKKGRKAVIAW					
Syn-safencin-29	AGKEKWRQYLKNEIKKKGRKAVIAW					
Syn-safencin-30	AGKETWRKYLKNEIKKKGRKAVIAW					
Syn-safencin-31	AGKETWRQKLKNEIKKKGRKAVIAW					
Syn-safencin-32	AGKEKWRKKLKNEIKKKGRKAVIAW	<20	4.62 ± 0.13	4.33		
Syn-safencin-33	AGKETIRQYWKNEIKKKGRKAVIAW					
Syn-safencin-34	AGKKTIRQYWKNEIKKKGRKAVIAW					
Syn-safencin-35	AGKETIRQYWKNKIKKKGRKAVIAW	<20	0.29 ± 0.10	68.03		
Syn-safencin-36	AGKKTIRQYWKNKIKKKGRKAVIAW	<20	1.29 ± 0.12	15.46		
Syn-safencin-37	AGKEKIRQYWKNEIKKKGRKAVIAW	<20	5.21 ± 0.17	3.84		
Syn-safencin-38	AGKETIRKYWKNEIKKKGRKAVIAW					
Syn-safencin-39	AGKETIRQKWKNEIKKKGRKAVIAW	<20	0.72 ± 0.04	27.89		
Syn-safencin-40	AGKEKIRKKWKNEIKKKGRKAVIAW	<20	2.12 ± 0.09	9.42		
Syn-safencin-41	AGKETIRQYLKNEWKKKGRKAVIAW					
Syn-safencin-42	AGKKTIRQYLKNEWKKKGRKAVIAW					
Syn-safencin-43	AGKETIRQYLKNKWKKKGRKAVIAW	<20	11.21 ± 0.20	1.78		

Syn-safencin-44	AGKKTIRQYLKNKWKKKGRKAVIAW	<20	0.63 ± 0.07	31.75		
Syn-safencin-45	AGKEKIRQYLKNEWKKKGRKAVIAW					
Syn-safencin-46	AGKETIRKYLKNEWKKKGRKAVIAW					
Syn-safencin-47	AGKETIRQKLKNEWKKKGRKAVIAW					
Syn-safencin-48	AGKEKIRKKLKNEWKKKGRKAVIAW	<20	1.75 ± 0.06	11.46		
Syn-safencin-49	AGKETIRQYLKNEIKKKWRKAVIAW					
Syn-safencin-50	AGKKTIRQYLKNEIKKKWRKAVIAW					
Syn-safencin-51	AGKETIRQYLKNKIKKKWRKAVIAW	<20	6.36 ± 0.10	3.15		
Syn-safencin-52	AGKKTIRQYLKNKIKKKWRKAVIAW	<20	0.58 ± 0.05	34.60		
Syn-safencin-53	AGKEKIRQYLKNEIKKKWRKAVIAW					
Syn-safencin-54	AGKETIRKYLKNEIKKKWRKAVIAW					
Syn-safencin-55	AGKETIRQKLKNEIKKKWRKAVIAW					
Syn-safencin-56	AGKEKIRKKLKNEIKKKWRKAVIAW					
Syn-safencin-57	AGKETIRQYLKNEIKKKGRKWVIAW					
Syn-safencin-58	AGKKTIRQYLKNEIKKKGRKWVIAW					
Syn-safencin-59	AGKETIRQYLKNKIKKKGRKWVIAW					
Syn-safencin-60	AGKKTIRQYLKNKIKKKGRKWVIAW	<20	1.31 ± 0.05	15.22		
Syn-safencin-61	AGKEKIRQYLKNEIKKKGRKWVIAW	<20	3.56 ± 0.10	5.62		
Syn-safencin-62	AGKETIRKYLKNEIKKKGRKGVIAW					
Syn-safencin-63	AGKETIRQKLKNEIKKKGRKWVIAW					
Syn-safencin-64	AGKEKIRKKLKNEIKKKGRKWVIAW	<20	0.33 ± 0.09	61.35		
Syn-safencin-65	AGKETIRQYLKNEIKKKGRKAWIAW	<20	1.01 ± 0.10	20.08		
Syn-safencin-66	AGKKTIRQYLKNEIKKKGRKAWIAW	>20	3.64 ± 0.10	5.49		
Syn-safencin-67	AGKETIRQYLKNKIKKKGRKAWIAW					
Syn-safencin-68	AGKKTIRQYLKNKIKKKGRKAWIAW	<20	0.56 ± 0.09	35.46		
Syn-safencin-69	AGKEKIRQYLKNEIKKKGRKAWIAW	<20	3.37 ± 0.06	5.93		

Syn-safencin-70	AGKETIRKYLKNEIKKKGRKAWIAW					
Syn-safencin-71	AGKETIRQKLKNEIKKKGRKAWIAW					
Syn-safencin-72	AGKEKIRKKLKNEIKKKGRKAWIAW	>20	12.07 ± 0.09	1.66		
Syn-safencin-73	AGKETIRQYLKNEIKKKGRKAVWAW	>20	0.35 ± 0.08	56.50	3.35 ± 0.08	5.97
Syn-safencin-74	AGKKTIRQYLKNEIKKKGRKAVWAW	<20	3.51 ± 0.06	5.70		
Syn-safencin-75	AGKETIRQYLKNKIKKKGRKAVWAW					
Syn-safencin-76	AGKKTIRQYLKNKIKKKGRKAVWAW	<20	16.32 ± 0.10	1.23		
Syn-safencin-77	AGKEKIRQYLKNEIKKKGRKAVWAW	>20	0.39 ± 0.13	51.28	1.59 ± 0.04	12.59
Syn-safencin-78	AGKETIRKYLKNEIKKKGRKAVWAW	>20	0.94 ± 0.14	21.19	3.89 ± 0.06	5.14
Syn-safencin-79	AGKETIRQKLKNEIKKKGRKAVWAW					
Syn-safencin-80	AGKEKIRKKLKNEIKKKGRKAVWAW					
Syn-safencin-81	AGKETIRQYLKNEIKKKGRKAVIWW	<20	3.15 ± 0.17	6.36		
Syn-safencin-82	AGKKTIRQYLKNEIKKKGRKAVIWW	>20	3.75 ± 0.10	53.33	1.02 ± 0.07	19.55
Syn-safencin-83	AGKETIRQYLKNKIKKKGRKAVIWW					
Syn-safencin-84	AGKKTIRQYLKNKIKKKGRKAVIWW					
Syn-safencin-85	AGKEKIRQYLKNEIKKKGRKAVIWW					
Syn-safencin-86	AGKETIRKYLKNEIKKKGRKAVIWW					
Syn-safencin-87	AGKETIRQKLKNEIKKKGRKAVIWW					
Syn-safencin-88	AGKEKIRKKLKNEIKKKGRKAVIWW					
Syn-safencin-89	AWKETIRQYLKNEIKKKWRKAVIAW	<20	1.05 ± 0.14	19.01		
Syn-safencin-90	AWKKTIRQYLKNEIKKKWRKAVIAW	<20	2.60 ± 0.19	7.82		
Syn-safencin-91	AWKETIRQYLKNKIKKKWRKAVIAW	<20	0.60 ± 0.12	33.22		
Syn-safencin-92	AWKKTIRQYLKNKIKKKWRKAVIAW	<20	0.90 ± 0.19	22.22		

Syn-safencin-93	AWKEKIRQYLKNEIKKKWRKAVIAW	<20	2.56 ± 0.18	7.81		
Syn-safencin-94	AWKETIRKYLKNEIKKKWRKAVIAW	>20	1.17 ± 0.07	17.05		
Syn-safencin-95	AWKETIRQKLKNEIKKKWRKAVIAW					
Syn-safencin-96	AWKEKIRKKLKNEIKKKWRKAVIAW					

Supplementary Table 5. Secondary screening results of Syn-sordellicin peptide library. Peptide sequence images were made using Geneious R6 version 6.1.8 for Windows. Syn-sordellicin-1 sequence is in full color, while syn-sordellicin-2 through -96 only have colored amino acids that differ from the sequence of syn-sordellicin-1. The highest concentration screened was 20μM. The values listed are the means of three replicates ± standard deviation (μM). Selectivity index (SI) is calculated as mean CC_{50}/IC_{50}. Axenic amastigote IC_{50} values were determined if below 20μM and those peptides were then screened against THP-1 macrophages. Cytotoxicity CC_{50} values were only determined if the peptide was effective against axenic amastigotes. Due to limited peptide availability, only the 10 peptide candidates with axenic amastigote SI values >20 were then screened in the intracellular amastigote model.

Name	Sequence	THP-1	Axenic Amastigotes		Intracellular Amastigotes	
		CC_{50} (μM)	IC_{50} (μM)	SI	IC_{50} (μM)	SI
Syn-sordellicin-1	AGRQTIKAYLRREIRKRGRKAVIAW		>20			
Syn-sordellicin-2	AGRQTIAKYLRREIRKRGRKAVIAW	<20	0.27 ± 0.18	74.07		
Syn-sordellicin-3	AGTQRIKAYLRREIRKRGRKAVIAW	<20	1.99 ± 0.13	10.04		
Syn-sordellicin-4	AGRQTIKAELRRYIRKRGRKAVIAW					
Syn-sordellicin-5	AGRQTIKAYLRREIRKVGRKARIAW					
Syn-sordellicin-6	AGRQTIKAYLRREIRKRGRIAVKAW	>20	14.25 ± 0.17	1.40		
Syn-sordellicin-7	AGTQRIAKELRRYIRKVGRIARKAW	<20	0.32 ± 0.07	62.89		
Syn-sordellicin-8	AGTNRIAKELRRYIRKVGRIARKAW					
Syn-sordellicin-9	WGRQTIKAYLRREIRKRGRKAVIAW					
Syn-sordellicin-10	WGRQTIAKYLRREIRKRGRKAVIAW	<20	0.82 ± 0.08	24.42		
Syn-sordellicin-11	WGTQRIKAYLRREIRKRGRKAVIAW					
Syn-sordellicin-12	WGRQTIKAELRRYIRKRGRKAVIAW					
Syn-sordellicin-13	WGRQTIKAYLRREIRKVGRKARIAW					
Syn-sordellicin-14	WGRQTIKAYLRREIRKRGRIAVKAW	<20	9.16 ± 0.13	2.18		
Syn-sordellicin-15	WGTQRIAKELRRYIRKVGRIARKAW					
Syn-sordellicin-16	WGTNRIAKELRRYIRKVGRIARKAW					
Syn-sordellicin-17	AWRQTIKAYLRREIRKRGRKAVIAW					
Syn-sordellicin-18	AWRQTIAKYLRREIRKRGRKAVIAW		4.90 ± 0.11			
Syn-sordellicin-19	AWTQRIKAYLRREIRKRGRKAVIAW	>20	12.98 ± 0.13	1.54		
Syn-sordellicin-20	AWRQTIKAELRRYIRKRGRKAVIAW					
Syn-sordellicin-21	AWRQTIKAYLRREIRKVGRKARIAW					
Syn-sordellicin-22	AWRQTIKAYLRREIRKRGRIAVKAW					
Syn-sordellicin-23	AWTQRIAKELRRYIRKVGRIARKAW	<20	0.25 ± 0.15	79.68		

Syn-sordellicin-24	AWTNRIAKELRRYIRKVGRIARKAW	<20	1.30 ± 0.05	15.35		
Syn-sordellicin-25	AGRQTIKAYLRREIRKRWRKAVIAW					
Syn-sordellicin-26	AGRQTIAKYLRREIRKRWRKAVIAW					
Syn-sordellicin-27	AGTQRIKAYLRREIRKRWRKAVIAW					
Syn-sordellicin-28	AGRQTIKAELRRYIRKRWRKAVIAW					
Syn-sordellicin-29	AGRQTIKAYLRREIRKVWRKARIAW					
Syn-sordellicin-30	AGRQTIKAYLRREIRKRWRIAVKAW					
Syn-sordellicin-31	AGTQRIAKELRRYIRKVWRIARKAW	<20	4.74 ± 0.09	4.22		
Syn-sordellicin-32	AGTNRIAKELRRYIRKVWRIARKAW	<20	0.23 ± 0.14	88.50		
Syn-sordellicin-33	AGRQTIKWYLRREIRKRGRKAVIAW					
Syn-sordellicin-34	AGRQTIWKYLRREIRKRGRKAVIAW					
Syn-sordellicin-35	AGTQRIKWYLRREIRKRGRKAVIAW	<20	1.79 ± 0.05	11.15		
Syn-sordellicin-36	AGRQTIKWELRRYIRKRGRKAVIAW	>20	7.59 ± 0.07	2.63		
Syn-sordellicin-37	AGRQTIKWYLRREIRKVGRKARIAW					
Syn-sordellicin-38	AGRQTIKWYLRREIRKRGRIAVKAW					
Syn-sordellicin-39	AGTQRIWKELRRYIRKVGRIARKAW	<20	0.33 ± 0.07	59.88		
Syn-sordellicin-40	AGTNRIWKELRRYIRKVGRIARKAW	<20	2.75 ± 0.07	7.29		
Syn-sordellicin-41	AGRQWIKAYLRREIRKRGRKAVIAW					
Syn-sordellicin-42	AGRQWIAKYLRREIRKRGRKAVIAW					
Syn-sordellicin-43	AGWQRIKAYLRREIRKRGRKAVIAW		14.75 ± 0.09			
Syn-sordellicin-44	AGRQWIKAELRRYIRKRGRKAVIAW	<20	0.28 ± 0.07	71.94		
Syn-sordellicin-45	AGRQWIKAYLRREIRKVGRKARIAW					
Syn-sordellicin-46	AGRQWIKAYLRREIRKRGRIAVKAW					
Syn-sordellicin-47	AGWQRIAKELRRYIRKVGRIARKAW	<20	3.23 ± 0.08	6.18		
Syn-sordellicin-48	AGWNRIAKELRRYIRKVGRIARKAW	<20	8.28 ± 0.09	2.42		
Syn-sordellicin-49	AGRQTIKAYLRREIRKRGRKWVIAW					
Syn-sordellicin-50	AGRQTIAKYLRREIRKRGRKWVIAW					
Syn-sordellicin-51	AGTQRIKAYLRREIRKRGRKWVIAW	<20	0.29 ± 0.10	68.97		
Syn-sordellicin-52	AGRQTIKAELRRYIRKRGRKWVIAW	<20	1.24 ± 0.04	16.16		
Syn-sordellicin-53	AGRQTIKAYLRREIRKVGRKWRIAW					
Syn-sordellicin-54	AGRQTIKAYLRREIRKRGRIWVKAW					
Syn-sordellicin-55	AGTQRIAKELRRYIRKVGRIWRKAW					
Syn-sordellicin-56	AGTNRIAKELRRYIRKVGRIWRKAW					
Syn-sordellicin-57	AGRQTIKAYLRREIRKRGRKAVIWW					
Syn-sordellicin-58	AGRQTIAKYLRREIRKRGRKAVIWW					

Syn-sordellicin-59	AGTQRIKAYLRREIRKRGRKAVIWW					
Syn-sordellicin-60	AGRQTIKAELRRYIRKRGRKAVIWW	<20	1.38 ± 0.11	14.50		
Syn-sordellicin-61	AGRQTIKAYLRREIRKVGRKARIWW	<20	0.49 ± 0.07	40.73		
Syn-sordellicin-62	AGRQTIKAYLRREIRKRGRIAVKWW					
Syn-sordellicin-63	AGTQRIAKELRRYIRKVGRIARKWW					
Syn-sordellicin-64	AGTNRIAKELRRYIRKVGRIARKWW	<20	1.64 ± 0.12	12.17		
Syn-sordellicin-65	AGRQTIKAYLRREIRKRKRKAVIAW	<20	10.39 ± 0.17	1.92		
Syn-sordellicin-66	AGRQTIAKYLRREIRKRKRKAVIAW					
Syn-sordellicin-67	AGTQRIKAYLRREIRKRKRKAVIAW					
Syn-sordellicin-68	AGRQTIKAELRRYIRKRKRKAVIAW					
Syn-sordellicin-69	AGRQTIKAYLRREIRKVKRKARIAW	<20	0.87 ± 0.11	23.07		
Syn-sordellicin-70	AGRQTIKAYLRREIRKRKRIAVKAW					
Syn-sordellicin-71	AGTQRIAKELRRYIRKVKRIARKAW					
Syn-sordellicin-72	AGTNRIAKELRRYIRKVKRIARKAW					
Syn-sordellicin-73	AGRQTIKAYLRREIRKRGRKKVIAW	<20	4.07 ± 0.09	4.91		
Syn-sordellicin-74	AGRQTIAKYLRREIRKRGRKKVIAW					
Syn-sordellicin-75	AGTQRIKAYLRREIRKRGRKKVIAW					
Syn-sordellicin-76	AGRQTIKAELRRYIRKRGRKKVIAW	<20	12.48 ± 0.09	1.60		
Syn-sordellicin-77	AGRQTIKAYLRREIRKVGRKKRIAW					
Syn-sordellicin-78	AGRQTIKAYLRREIRKRGRIKVKAW					
Syn-sordellicin-79	AGTQRIAKELRRYIRKVGRIKRKAW					
Syn-sordellicin-80	AGTNRIAKELRRYIRKVGRIKRKAW	<20	1.20 ± 0.10	16.74		
Syn-sordellicin-81	AGRQTIKAYLRREIRKRGRKAVIKW					
Syn-sordellicin-82	AGRQTIAKYLRREIRKRGRKAVIKW					
Syn-sordellicin-83	AGTQRIKAYLRREIRKRGRKAVIKW					
Syn-sordellicin-84	AGRQTIKAELRRYIRKRGRKAVIKW					
Syn-sordellicin-85	AGRQTIKAYLRREIRKVGRKARIKW					
Syn-sordellicin-86	AGRQTIKAYLRREIRKRGRIAVKKW					
Syn-sordellicin-87	AGTQRIAKELRRYIRKVGRIARKKW					
Syn-sordellicin-88	AGTNRIAKELRRYIRKVGRIARKKW					
Syn-sordellicin-89	AKRQTIKAYLRREIRKRGRKAVIAW	<20	5.21 ± 0.11	3.84		
Syn-sordellicin-90	AKRQTIAKYLRREIRKRGRKAVIAW					
Syn-sordellicin-91	AKTQRIKAYLRREIRKRGRKAVIAW					
Syn-sordellicin-92	AKRQTIKAELRRYIRKRGRKAVIAW					
Syn-sordellicin-93	AKRQTIKAYLRREIRKVGRKARIAW					
Syn-sordellicin-94	AKRQTIKAYLRREIRKRGRIAVKAW					
Syn-sordellicin-95	AKTQRIAKELRRYIRKVGRIARKAW					
Syn-sordellicin-96	AKTNRIAKELRRYIRKVGRIARKAW					

87

Supplementary Table 6. Secondary screening results of Syn-xiamencin peptide library. Peptide sequence images were made using Geneious R6 version 6.1.8 for Windows. Syn-xiamencin-1 sequence is in full color, while syn-xiamencin-2 through -96 only have colored amino acids that differ from the sequence of syn-xiamencin-1. The highest concentration screened was 20µM. The values listed are the means of three replicates ± standard deviation (µM). Selectivity index (SI) is calculated as mean CC_{50}/IC_{50}. Axenic amastigote IC_{50} values were determined if below 20µM and those peptides were then screened against THP-1 macrophages. Cytotoxicity CC_{50} values were only determined if the peptide was effective against axenic amastigotes. Due to limited peptide availability, only the 10 peptide candidates with axenic amastigote SI values >20 were then screened in the intracellular amastigote model.

Name	Sequence	THP-1	Axenic Amastigotes		Intracellular Amastigotes	
		CC_{50} (µM)	IC_{50} (µM)	SI	IC_{50} (µM)	SI
Syn-xiamencin-1	AGRQALTLYLKEELRKRGKKAFIAW		>20			
Syn-xiamencin-2	AGAQRLTLYLKEELRKRGKKAFIAW	<20	0.92 ± 0.21	21.79		
Syn-xiamencin-3	AGRQALTLELKEYLRKRGKKAFIAW	>20	1.26 ± 0.17	15.87		
Syn-xiamencin-4	AGRQALTKYLLEELRKRGKKAFIAW	<20	4.73 ± 0.16	4.23		
Syn-xiamencin-5	AGRQALTLYLKEELRKFGKKARIAW					
Syn-xiamencin-6	AGRQALTLYLKEELRKRGKIAFKAW	<20	0.61 ± 0.08	32.63		
Syn-xiamencin-7	AGAQRLTKELLEYLRKFGKIARKAW	<20	1.47 ± 0.18	13.60		
Syn-xiamencin-8	AGANRLTKELLEYLRKFGKIARKAW	<20	3.04 ± 0.12	6.59		
Syn-xiamencin-9	WGRQALTLYLKEELRKRGKKAFIAW					
Syn-xiamencin-10	WGAQRLTLYLKEELRKRGKKAFIAW	<20	0.94 ± 0.12	21.25		
Syn-xiamencin-11	WGRQALTLELKEYLRKRGKKAFIAW	<20	1.90 ± 0.19	10.51		
Syn-xiamencin-12	WGRQALTKYLLEELRKRGKKAFIAW	<20	1.76 ± 0.07	11.36		
Syn-xiamencin-13	WGRQALTLYLKEELRKFGKKARIAW					

Syn-xiamencin-14	WGRQALTLYLKEELRKRGKIAFKAW	<20	0.38 ± 0.03	52.91		
Syn-xiamencin-15	WGAQRLTKELLEYLRKFGKIARKAW	<20	0.43 ± 0.08	46.84		
Syn-xiamencin-16	WGANRLTKELLEYLRKFGKIARKAW	<20	1.55 ± 0.10	12.91		
Syn-xiamencin-17	AGRQALTLYLKEELRKRWKKAFIAW	<20	0.39 ± 0.03	51.41		
Syn-xiamencin-18	AGAQRLTLYLKEELRKRWKKAFIAW	<20	0.65 ± 0.06	30.86		
Syn-xiamencin-19	AGRQALTLELKEYLRKRWKKAFIAW	<20	2.15 ± 0.11	9.30		
Syn-xiamencin-20	AGRQALTKYLLEELRKRWKKAFIAW	<20	0.47 ± 0.05	42.55		
Syn-xiamencin-21	AGRQALTLYLKEELRKFWKKARIAW	<20	0.59 ± 0.08	33.90		
Syn-xiamencin-22	AGRQALTLYLKEELRKRWKIAFKAW	<20	2.21 ± 0.11	9.07		
Syn-xiamencin-23	AGAQRLTKELLEYLRKFWKIARKAW	<20	1.14 ± 0.10	17.54		
Syn-xiamencin-24	AGANRLTKELLEYLRKFWKIARKAW	<20	1.63 ± 0.05	12.31		
Syn-xiamencin-25	AWRQALTLYLKEELRKRGKKAFIAW					
Syn-xiamencin-26	AWAQRLTLYLKEELRKRGKKAFIAW					
Syn-xiamencin-27	AWRQALTLELKEYLRKRGKKAFIAW					
Syn-xiamencin-28	AWRQALTKYLLEELRKRGKKAFIAW					
Syn-xiamencin-29	AWRQALTLYLKEELRKFGKKARIAW					
Syn-xiamencin-30	AWRQALTLYLKEELRKRGKIAFKAW					
Syn-xiamencin-31	AWAQRLTKELLEYLRKFGKIARKAW	<20	4.82 ± 0.09	4.15		
Syn-xiamencin-32	AWANRLTKELLEYLRKFGKIARKAW	<20	0.89 ± 0.10	22.47		
Syn-xiamencin-33	AGRQALTLYLKEELRKRGKKWFIAW	<20	2.66 ± 0.10	7.53		

Syn-xiamencin-34	AGAQRLTLYLKEELRKRGKKWFIAW					
Syn-xiamencin-35	AGRQALTLELKEYLRKRGKKWFIAW	<20	7.37 ± 0.08	5.93		
Syn-xiamencin-36	AGRQALTKYLLEELRKRGKKWFIAW	<20	1.52 ± 0.09	13.14		
Syn-xiamencin-37	AGRQALTLYLKEELRKFGKKWRIAW	<20	3.71 ± 0.11	5.39		
Syn-xiamencin-38	AGRQALTLYLKEELRKRGKIWFKAW					
Syn-xiamencin-39	AGAQRLTKELLEYLRKFGKIWRKAW	<20	6.81 ± 0.07	2.94		
Syn-xiamencin-40	AGANRLTKELLEYLRKFGKIWRKAW	<20	1.31 ± 0.11	15.27		
Syn-xiamencin-41	AGRQALWLYLKEELRKRGKKAFIAW	>20	2.73 ± 0.15	7.33		
Syn-xiamencin-42	AGAQRLWLYLKEELRKRGKKAFIAW	>20	6.83 ± 0.09	2.93		
Syn-xiamencin-43	AGRQALWLELKEYLRKRGKKAFIAW	>20	3.54 ± 0.08	5.65		
Syn-xiamencin-44	AGRQALWKYLLEELRKRGKKAFIAW	<20	1.18 ± 0.06	16.91		
Syn-xiamencin-45	AGRQALWLYLKEELRKFGKKARIAW	<20	6.13 ± 0.09	3.26		
Syn-xiamencin-46	AGRQALWLYLKEELRKRGKIAFKAW	<20	11.84 ± 0.09	1.69		
Syn-xiamencin-47	AGAQRLWKELLEYLRKFGKIARKAW	<20	2.68 ± 0.03	7.46		
Syn-xiamencin-48	AGANRLWKELLEYLRKFGKIARKAW	<20	2.18 ± 0.03	9.17		
Syn-xiamencin-49	AGRQWLTLYLKEELRKRGKKAFIAW	<20	9.22 ± 0.06	2.17		
Syn-xiamencin-50	AGWQRLTLYLKEELRKRGKKAFIAW	<20	3.74 ± 0.05	5.35		

Syn-xiamencin-51	AGRQWLTLELKEYLRKRGKKAFIAW	<20	3.66 ± 0.04	5.46		
Syn-xiamencin-52	AGRQWLTKYLLEELRKRGKKAFIAW	>20	5.23 ± 0.06	3.82		
Syn-xiamencin-53	AGRQWLTLYLKEELRKFGKKARIAW	<20	14.83 ± 0.14	1.35		
Syn-xiamencin-54	AGRQWLTLYLKEELRKRGKIAFKAW	<20	7.79 ± 0.06	2.57		
Syn-xiamencin-55	AGWQRLTKELLEYLRKFGKIARKAW	<20	2.42 ± 0.05	8.26		
Syn-xiamencin-56	AGWNRLTKELLEYLRKFGKIARKAW	<20	2.42 ± 0.04	8.25		
Syn-xiamencin-57	AGRQALTLYLKEELRKRGKKAFIWW					
Syn-xiamencin-58	AGAQRLTLYLKEELRKRGKKAFIWW					
Syn-xiamencin-59	AGRQALTLELKEYLRKRGKKAFIWW					
Syn-xiamencin-60	AGRQALTKYLLEELRKRGKKAFIWW	<20	4.79 ± 0.03	4.17		
Syn-xiamencin-61	AGRQALTLYLKEELRKFGKKARIWW	<20	5.66 ± 0.06	3.54		
Syn-xiamencin-62	AGRQALTLYLKEELRKRGKIAFKWW	<20	3.52 ± 0.04	5.69		
Syn-xiamencin-63	AGAQRLTKELLEYLRKFGKIARKWW					
Syn-xiamencin-64	AGANRLTKELLEYLRKFGKIARKWW	<20	1.77 ± 0.03	11.31		
Syn-xiamencin-65	AGRQALTLYLKEELRKRKKKAFIAW					
Syn-xiamencin-66	AGAQRLTLYLKEELRKRKKKAFIAW					
Syn-xiamencin-67	AGRQALTLELKEYLRKRKKKAFIAW					
Syn-xiamencin-68	AGRQALTKYLLEELRKRKKKAFIAW					
Syn-xiamencin-69	AGRQALTLYLKEELRKFKKKARIAW	<20	5.86 ± 0.05	3.41		
Syn-xiamencin-70	AGRQALTLYLKEELRKRKKIAFKAW	<20	2.28 ± 0.03	8.76		
Syn-xiamencin-71	AGAQRLTKELLEYLRKFKKIARKAW					
Syn-xiamencin-72	AGANRLTKELLEYLRKFKKIARKAW	<20	4.25 ± 0.04	4.70		

Syn-xiamencin-73	AGRQALKLYLKEELRKRGKKAFIAW	<20	3.91 ± 0.07	5.12		
Syn-xiamencin-74	AGAQRLKLYLKEELRKRGKKAFIAW					
Syn-xiamencin-75	AGRQALKLELKEYLRKRGKKAFIAW					
Syn-xiamencin-76	AGRQALKKYLLEELRKRGKKAFIAW	<20	3.64 ± 0.05	5.49		
Syn-xiamencin-77	AGRQALKLYLKEELRKFGKKARIAW					
Syn-xiamencin-78	AGRQALKLYLKEELRKRGKIAFKAW					
Syn-xiamencin-79	AGAQRLKKELLEYLRKFGKIARKAW					
Syn-xiamencin-80	AGANRLKKELLEYLRKFGKIARKAW	<20	14.26 ± 0.12	1.40		
Syn-xiamencin-81	AGRQKLTLYLKEELRKRGKKAFIAW					
Syn-xiamencin-82	AGKQRLTLYLKEELRKRGKKAFIAW					
Syn-xiamencin-83	AGRQKLTLELKEYLRKRGKKAFIAW					
Syn-xiamencin-84	AGRQKLTKYLLEELRKRGKKAFIAW					
Syn-xiamencin-85	AGRQKLTLYLKEELRKFGKKARIAW					
Syn-xiamencin-86	AGRQKLTLYLKEELRKRGKIAFKAW					
Syn-xiamencin-87	AGKQRLTKELLEYLRKFGKIARKAW					
Syn-xiamencin-88	AGKNRLTKELLEYLRKFGKIARKAW					
Syn-xiamencin-89	AKRQALTLYLKEELRKRGKKAFIAW	<20	7.30 ± 0.07	2.74		
Syn-xiamencin-90	AKAQRLTLYLKEELRKRGKKAFIAW	<20	8.92 ± 0.08	2.24		
Syn-xiamencin-91	AKRQALTLELKEYLRKRGKKAFIAW					
Syn-xiamencin-92	AKRQALTKYLLEELRKRGKKAFIAW					
Syn-xiamencin-93	AKRQALTLYLKEELRKFGKKARIAW					
Syn-xiamencin-94	AKRQALTLYLKEELRKRGKIAFKAW					
Syn-xiamencin-95	AKAQRLTKELLEYLRKFGKIARKAW	<20	4.85 ± 0.03	4.12		
Syn-xiamencin-96	AKANRLTKELLEYLRKFGKIARKAW					
